# A multi-source data-based modelling study on brucellosis transmission risk analysis and control strategies in Zhejiang Province, China

**DOI:** 10.1186/s12879-026-12787-9

**Published:** 2026-02-20

**Authors:** Juan Li, Wei Jiang, Junhui Zhang, Lingyan Zhao, Shubo Li, Lu Gao, Siqi Sun, Hongli Zhang

**Affiliations:** 1 School of Computer Science and Technology (School of Artificial Intelligence), Zhejiang Sci-Tech University, Hangzhou, 310018 P.R. China; 2 Zhejiang Provincial Center for Animal Disease Control and Prevention, Hangzhou, 310020 P.R. China; 3 Liaoning Center for Animal Disease Control and Prevention, Shenyang, 110164 P.R. China; 4 School of Animal Science and Technology (School of Animal Medicine), Huazhong Agricultural University, Wuhan, 430070 P. R. China; 5 China Animal Health and Epidemiology Center, Qingdao, 266032 P.R. China

**Keywords:** Brucellosis, Non-autonomous dynamical model, Data-driven approach, Quantitative assessment, Prevention and control

## Abstract

**Background:**

Brucellosis presents a severe challenge to public health due to its complex transmission mechanism. In China, regions are categorized into three types based on the number of new human brucellosis cases or provinces affected by animal outbreaks, with tailored control strategies adopted accordingly. Currently, most research on brucellosis transmission modeling focuses on Type I regions. However, due to regional variations in farming practices, influencing factors, and control measures, existing studies are highly region-specific, restricting their direct application to Type II regions such as Zhejiang Province. Notably, annual brucellosis outbreaks in Zhejiang Province are worsening, highlighting the need for targeted research.

**Methods:**

In this study, a non-autonomous patch dynamical model was developed for Zhejiang Province, incorporating the “live sheep - mutton - human” transmission chain. The effective reproduction number was defined to calculate infection metrics for pathogen transmission in three pathways: sheep-to-sheep, sheep-to-human, and mutton-to-human. Multi-source data, including demographics, livestock farming, and meat transportation data from 2018 to 2024, were integrated. Key drivers of brucellosis transmission were explored through parameter estimation, sensitivity analysis, and multi-scenario numerical simulations.

**Results:**

The findings indicated a significant positive correlation between the number of live sheep imported from Region I and human brucellosis incidence rates, suggesting that imported infections have become the primary challenge for brucellosis control in Zhejiang Province. The baseline projected incidence in Zhejiang Province is expected to remain 0.3 per 100,000 by 2026 under current interventions. A 71.4% increase in human incidence (peaking at 0.6 per 100,000) in 2025 would occur if live sheep imports from other provinces were doubled, underscoring the risk of external introduction. Conversely, enhancing transport detection by 15% could reduce incidence below 0.2 per 100,000, while an integrated strategy combining improved culling, transport detection, and consumption monitoring could lower incidence to 0.1 per 100,000, representing a 66.7% reduction. Similarly, reducing both live sheep and mutton imports by 50% would decrease incidence by 40%, preventing an estimated 91 infections. Furthermore, improving comprehensive protection awareness by 50% is projected to reduce incidence to below 0.15 per 100,000, corresponding to a 53.3% decrease by 2026. Under non-immunization intervention scenarios, surveillance gaps in the farming-transportation-consumption chains could lead to a significant expansion of the epidemic. Implementing an integrated strategy combining source quarantine (testing imported live sheep), process control (regular monitoring of farming environments), and end-point protection (personal protective measures) was found to substantially reduce incidence rates within two years.

**Conclusions:**

The results of this study suggest that strengthening the monitoring of the inter-provincial live animal transportation surveillance network is crucial, as it can facilitate early warning of brucellosis transmission risks. This highlights the importance of targeted measures to address imported infections and enhance surveillance across the entire transmission chain for effective brucellosis control in Type II regions like Zhejiang Province.

## Introduction

Brucellosis, an acute and chronic zoonotic infectious disease caused by Brucella that is listed as a legally reportable and monitored infectious disease by the World Health Organization (WHO), affects over 60 species of domestic animals (including poultry), and wildlife with high cross-species transmission capacity, thereby posing a significant threat to both human health and livestock production [1–3]. This disease rages globally, spreading across 170 countries and regions, with an incidence of up to 1.6 - 2.1 million new human cases annually [4]. It seriously impacts the public health systems, weakens the production efficiency of animal husbandry, and has a profound impact on international trade. It is estimated that the direct agricultural economic losses caused by brucellosis globally each year are nearly 3 billion yuan, and the comprehensive economic losses have even soared to billions of US dollars [5].

**Fig. 1:**
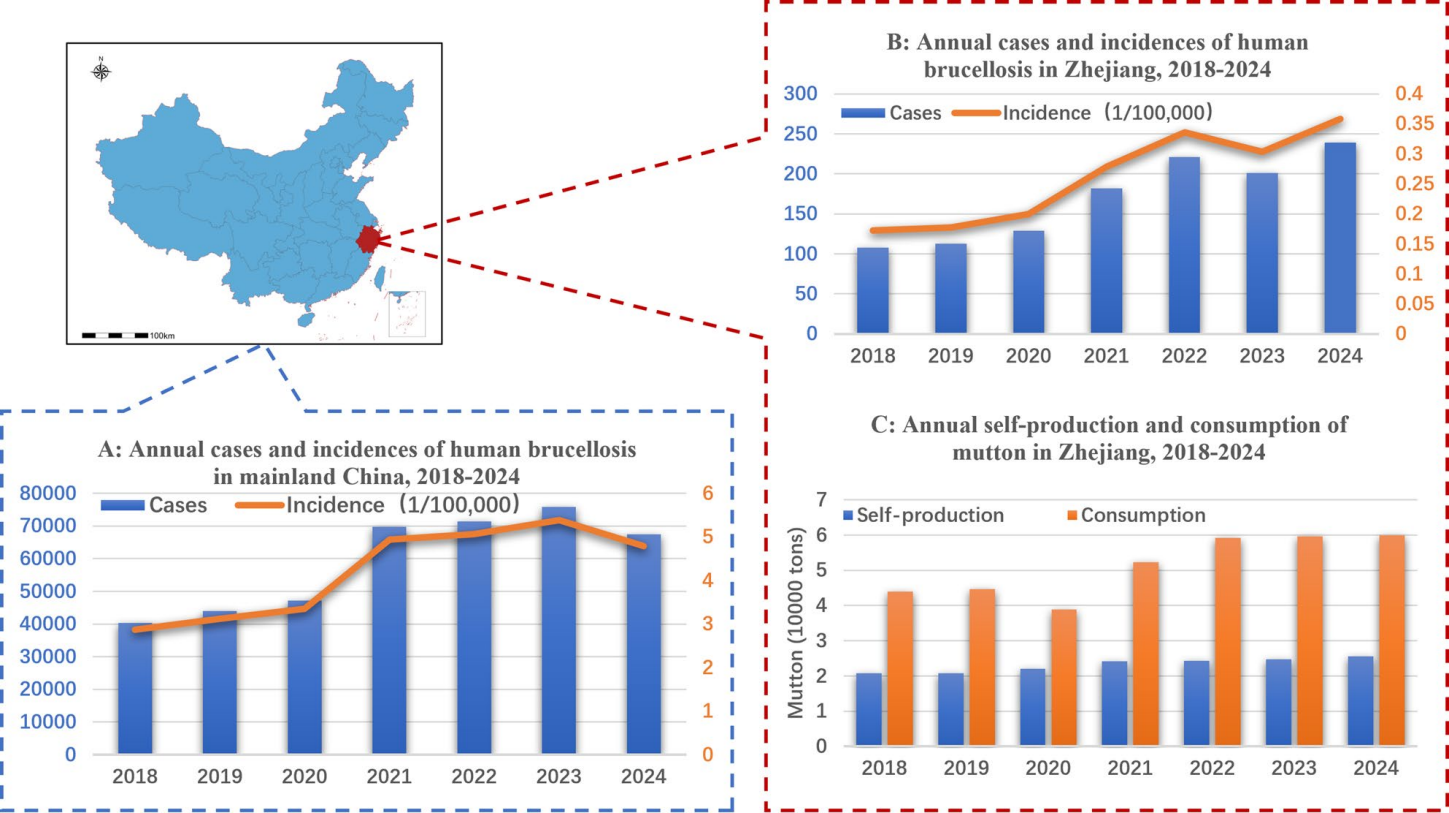
Number of human brucellosis cases and incidences in mainland China and Zhejiang Province, as well as the trend of changes in the quantity and consumption of self-produced mutton in Zhejiang (2018-2024). Data source details are in Subsection: Data resource

In China, brucellosis is classified as a Class II animal epidemic and a Class B infectious disease [6]. In recent years, the epidemic situation of brucellosis in the country has worsened. The number of human cases has surged from 37,911 in 2018 to 75,858 in 2023, the incidence rates increased from 2.72 per 100,000 (95% CI: 2.69–2.75) in 2018 to 5.38 per 100,000 (95% CI: 5.34–5.42) in 2023, with an average annual growth rate of 14.9% (Fig. 1.A). Geographically across the country, the epidemic shows the distinctive characteristics of “high incidence in the north and spreading in the south-west” [7]. As a representative case of epidemic evolution in southern China, Zhejiang Province has witnessed the spread of brucellosis to 10 prefecture-level cities since the first outbreak, which was triggered by the introduction of infected dairy cows in the 1950s [8]. To address the threat of brucellosis, the Ministry of Agriculture and Rural Affairs of all People’s of China (MOA) issued the “National Brucellosis Prevention and Control Plan (2016–2020)” in 2016 [9]. This plan designated Zhejiang Province as a Type II region and implemented a targeted monitoring-purification prevention and control strategy. However, systemic challenges, including the widespread distribution of infection sources, inadequate prevention and control funding, and deficiencies in the grassroots epidemic prevention system-have contributed to a continuing rise in the number of human brucellosis cases in the province from 108 in 2018 to 227 in 2024. In 2022, the number of reported cases reached 221, marking a historical high with an incidence rate of 0.336 per 100,000 population, see Fig. 1.B. The persistent upward trend in brucellosis cases has prompted MOA to sequentially introduce a national plan [10] and Zhejiang also launched a “Five-Year Action Program” with multi-tiered prevention and control framework aiming at controlling the Brucellosis in livestock, most importantly to control the human brucellosis incidence below 0.5 per 100,000 population [11, 12]. It is noteworthy that despite the well-defined and implemented prevention and control strategies, the efficacy of brucellosis mitigation remains contingent on continuous monitoring and scientific evaluation. This necessity arises from the transmission mechanisms which are intricately shaped by temporal and spatial variations, as well as the complex interplay of natural and social factors. For instance, seasonal variations in lambing seasons can concentrate environmental contamination (a natural factor), while long-distance livestock trade for seasonal festivals (a social-economic factor) can rapidly disseminate the pathogen across regions [13].

Currently, the prevention and control of brucellosis in China is at a critical stage of strategic transition with Zhejiang emerged as a notable exemplar. With its expansive territory and high population density, the province faces land resource constraints and the self-sufficiency rate in cattle and sheep production remains below 40% (as depicted in Fig. 1.C), leading to heavily reliance on external meat products. These factors collectively amplify the complexity and challenges inherent to brucellosis mitigation. It is essential to investigate by leveraging multi-source data to conduct an in-depth analysis of brucellosis transmission dynamics in Zhejiang, to systematically evaluate the impact of multifaceted determinants and the actual efficacy of current interventions, thereby enabling improvement of control strategies to overcome the bottlenecks in brucellosis management.

Considering the inherent non-experimental repeatability constraints in infectious disease transmission dynamics, mathematical modeling has emerged a pivotal theoretical framework [14] for exploring disease transmission mechanisms and evaluating the efficacy of prevention and control strategies [15, 16]. In the field of brucellosis research, significant progress has been achieved through studies such as [14, 17–23] and [8, 24–33], with systematic reviews presented by [13] and [34]. To investigate the impact of cross-regional transportation and trade of livestock on brucellosis transmission, several patch dynamic models have been developed, exploring in detail the interconnectedness of brucellosis prevalence across multiple regions [30, 35, 36]. However, existing modelling studies of brucellosis predominantly focus on Class I regions of China. Few quantitative models have been developed for Type II provinces with high import dependency. Consequently, findings from Class I regions have limited applicability to regions like Zhejiang Province. Furthermore, the scarcity of data and the absence of quantitative analysis pose challenges in accurately identifying and assessing the specific impacts of dominant factors associate with the brucellosis transmission in the province. This impedes the precision of epidemic trend predictions and the refinement of prevention and control strategies, resulting in a lack of targeted and forward-thinking policy adjustments.

This study aligns with the guiding principles outlined in the Five-Year Action Plan [11, 12] of Zhejiang, we will account for the substantial influence of inter-provincial transportation on brucellosis transmission by delineating regions with Zhejiang Province as a distinct entity and the remainder of the country as one other to establish a cross-regional patch model. Utilizing this model, we will conduct a quantitative evaluation of the ongoing continuous monitoring and purification measures for livestock brucellosis in the province, adhering to non-immunization principles. Additionally, the study will further evaluate the effects of detection efficiency in breeding, transportation, and consumption processes on the transmission risk of brucellosis. In light of the escalating demand for mutton consumption, we will forecast the prevalence trend of human brucellosis which will offer robust support for decision making. Furthermore, multiple prevention and control strategy scenario simulations will be presented and compared for an optimization recommendation for enhancing the control and mitigation capabilities of brucellosis. This study will not only furnish a robust theoretical framework and empirical data for the development of precise and effective brucellosis prevention and control strategies in Zhejiang Province.

## Materials and Methods

### Data resource

In this study, we collected and integrated data resources from multiple reputable sources to study the transmission mechanisms and assess the risk of brucellosis in Zhejiang Province, refer to Table 1 for full details. The foundation of our analysis relies on three groups of data:

**Table 1:** Data summary table

Data sources	Temporal coverage	URLs
Annual national human brucellosis case	2018–2024	https://www.chinacdc.cn/jksj/jksj01/202410/t20241010_301346.html.
Provincial-level human brucellosis data for Zhejiang Province	2018–2024	https://wsjkw.zj.gov.cn/col/col1229123469/index.html
Sheep inventory, annual slaughter volume, and average meat yield per sheep (both national and provincial)	2018–2024	http://60.16.24.131/CSYDMirror/area/yearbook/Single/N2023030190?z=D15 http://zjzd.stats.gov.cn/dcsj/ndsj_2174/2023_ndsj/lscmyqk/202401/t20240123_110542.html https://www.stats.gov.cn/sj/sjjd/202302/t20230202_1896736.html https://www.stats.gov.cn/sj/sjjd/202401/t20240118_1946695.html
Demographic indictors (total population, births, deaths) for China and Zhejiang Province	2018–2024	https://www.stats.gov.cn/sj/ndsj/2023/indexch.htm https://tjj.zj.gov.cn/art/2023/10/16/art_1525563_58960915.html https://www.stats.gov.cn/sj/zxfb/202402/t20240228_1947915.html https://www.stats.gov.cn/sj/zxfb/202502/t20250228_1958817.html https://tjj.zj.gov.cn/art/2025/3/1/art_1229129205_5469687.html https://tjj.zj.gov.cn/art/2023/2/22/art_1229129205_5070151.html
Annual slaughter quantity	2018–2024	https://www.stats.gov.cn/sj/ndsj/2023/indexch.htm
The average meat yield per sheep	2018–2024	http://zjzd.stats.gov.cn/dcsj/ndsj_2174/2023_ndsj/lscmyqk/202401/t20240123_110542.html
Annual permanent population	2018–2024	https://www.shujuku.org/china-animal-husbandry-and-veterinary-yearbook.html
The per capita mutton consumption	2018–2024	https://tjj.zj.gov.cn/art/2023/10/16/art_1525563_58960915.html

#### Human brucellosis cases

The human brucellosis case data used in this study are confirmed clinical cases. These cases were diagnosed strictly in accordance with the national diagnostic criteria for brucellosis, which require the combination of epidemiological exposure history, typical clinical manifestations, and positive results of both serological tests and etiological examinations.

- Annual national human brucellosis case records (2018-2024) were from the National Disease Control and Prevention Administration [37].
- Provincial-level human brucellosis data for Zhejiang Province (2018-2024) were sourced from the Health Commission of Zhejiang Province [38].

#### Livestock industry data

- Sheep inventory, annual slaughter volume, and average meat yield per sheep (both national and provincial) (2018-2022) were extracted from the “China Animal Husbandry and Veterinary Medicine Yearbook” [39].
- For 2023-2024, these livestock data was supplemented from the National Bureau of Statistics (NBS) of China [40–42].

#### Demographic and consumption indicators

- Demographic indictors (total population, births, deaths) for China and Zhejiang Province (2018-2022) form the “China Statistical Yearbook, 2023” [43] and “Zhejiang Statistical Yearbook, 2023” [44].
- For 2023-2024, national demographic updates were obtained from the “Statistical Bulletin of the People’s Republic of China on National Economic and Social Development” in 2023 and 2024 [45, 46], and the “Main Data Bulletin of Population of Zhejiang Province” in 2023 and 2024 [47, 48].

To elucidate potential transmission routes within Zhejiang, we estimated annual mutton imports (2018–2024) using the formula mutton imports (kg) = Total consumption (kg)− self-produced mutton (kg),

- Self-Produced mutton = annual slaughter quantity [43] (head)× average meat yield per sheep [49] (kg/head)
- Total consumption = annual permanent population [50] (Individual)× per capita mutton consumption [44] (kg/Individual).
- For example, using 2020 data: Self-Produced mutton = 1,299,000 (head) × 15.47 (kg/head) =  20,095,530 kg; Total consumption = 50,690,000 (individuals) × 0.9 (kg/individual) =  45,621,000 kg. Therefore, the estimated mutton import for 2020 was 45,621,000 kg − 20,095,530 kg = 25,525,470 kg.

These comprehensive datasets, derived from authoritative sources, provide a solid foundation for constructing dynamic models and estimating parameters in our analysis.

### Model formulation

Based on the available data, this study constructs a macro-scale patch-model brucellosis transmission dynamics model that takes mainland China as the research area and the month as the temporal unit, with Zhejiang Province designated as a single patch and the rest of China grouped into another patch. The model’s foundation revolves around three primary entities: the human population (${N_{hi}}$), the sheep population (${N_{si}}$), and mutton (${M_i}$), where $i = 1$ and $2$ represent the Region I and Region II, respectively. Prior to constructing the dynamic model, we established the following premises based on empirical realities:

1. Both human and sheep populations are treated as homogeneous, disregarding age variations among humans or breed and age disparities among sheep.
2. Given the absence of definitive evidence of human-to-human transmission of brucellosis [51], this transmission route is excluded from the model.
3. The model assumes that cross-regional transportation of live sheep or mutton is unidirectional, i.e., exclusively from Region I into Zhejiang Province. This assumption is underpinned by two key facts: Zhejiang’s mutton consumption far outstrips its self-sufficiency, driving inherent demand for external supply; And it is supported by formal trade statistics and livestock product circulation regulations. Though potential reverse flows via illegal or informal channels cannot be fully excluded, limited surveillance data show such unregulated trade is small-scale, sporadic, and has negligible impacts on the model-captured overall transmission dynamics.
4. Due to the lack of empirical data supporting continuous environmental pathogen monitoring, the model solely considers human infection routes through contact with infected sheep or consumption of contaminated mutton.

Drawing from these assumptions, we developed a two-patch model coupling sheep, humans, and mutton. This model was used to systematically examine the impacts of cross-provincial circulation of live sheep and their products on brucellosis transmission in Zhejiang Province. Employing a multi-factor coupling analysis framework, we will quantitatively assess the contribution of exogenous input factors to the local epidemic and forecast the degree of interference posed by this risk variable on the efficacy of existing prevention and control strategies. Additionally, this model features predictive capabilities, enabling a prediction of the brucellosis epidemic’s trajectory over the next two years, thereby offering crucial insights for prevention and control efforts. Specifically, we categorized the human population into two groups: susceptible (${S_{hi}}$) and infected (${I_{hi}}$), and similarly, the sheep population into susceptible (${S_{si}}$) and infected (${I_{si}}$), while mutton (${M_i}$) is treated as a single variable to underscore its role in transmission. The biological interpretations and units of all variables are provided in detail in Table 2.

**Table 2:** Table of state variables and their descriptions

Variables	Comments	Unit	Values	References
${{\bf{\it{S}}}_{{\bf{\it{s}}}1}}\left( {\bf{\it{t}}} \right)$	Number of susceptible sheep in Region I at time t	head	${S_{s1}}\left( 0 \right) = 2.9288\times 10^{8}$	Data
${{\bf{\it{I}}}_{{\bf{\it{s}}}1}}\left( {\bf{\it{t}}} \right)$	Number of infected sheep in Region I at time t	head	${I_{s1}}\left( 0 \right) = 4.9898\times 10^{6}$	Assumption
${{\bf{\it{M}}}_1}\left( {\bf{\it{t}}} \right)$	Quantity of mutton in Region I at time t	kg	${M_1}\left( 0 \right) = 6.8982\times 10^{10}$	Estimated
${{\bf{\it{S}}}_{{\bf{\it{h}}}1}}\left( {\bf{\it{t}}} \right)$	Number of susceptible humans in Region I at time t	Individual	${S_{h1}}\left( 0 \right) = 1.3400\times 10^{9}$	Assumption
${{\bf{\it{I}}}_{{\bf{\it{h}}}1}}\left( {\bf{\it{t}}} \right)$	Number of infected humans in Region I at time t	Individual	${I_{h1}}\left( 0 \right) = 5.9625\times 10^{4}$	Assumption
${{\bf{\it{S}}}_{{\bf{\it{s}}}2}}\left( {\bf{\it{t}}} \right)$	Number of susceptible sheep in Region II at time t	head	${S_{s2}}\left( 0 \right) = 1.2577\times 10^{6}$	Data
${{\bf{\it{I}}}_{{\bf{\it{s}}}2}}\left( {\bf{\it{t}}} \right)$	Number of infected sheep in Region II at time t	head	${I_{s2}}\left( 0 \right) = 1133$	Data
${{\bf{\it{M}}}_2}\left( {\bf{\it{t}}} \right)$	Quantity of mutton in Region II at time t	kg	${M_2}\left( 0 \right) = 1.7155\times 10^{7}$	Estimated
${{\bf{\it{S}}}_{{\bf{\it{h}}}2}}\left( {\bf{\it{t}}} \right)$	Number of susceptible humans in Region II at time t	Individual	${S_{h2}}\left( 0 \right) = 6.273\times 10^{7}$	Data
${{\bf{\it{I}}}_{{\bf{\it{h}}}2}}\left( {\bf{\it{t}}} \right)$	Number of infected humans in Region II at time t	Individual	${I_{h2}}\left( 0 \right) = 158$	Assumption

^1^Estimated: Values are estimated using the least squares method, based on the reported data on human brucellosis cases in two regions provided in Subsection: Data resource 2.1, as detailed in Subsection: Parameter estimation 3.1.

^2^Assumption: Values are assumed based on prior data, as detailed in Subsection: Parameter estimation 3.1.

^3^Data: Values are given based on official website, as detailed in Subsection: Data resource 2.1.

Guided by annual data from both regions, time-varying parameters are used to characterize the annual increase in sheep numbers ($${A_i}\left( t \right)$$), the sheep slaughter rate ($${\rho _i}\left( t \right)$$), the annual human population birth ($${B_i}(t$$)$$)$$, and the human population mortality rate ($${d_{hi}}\left( t \right)$$). Furthermore, the primary prevention and control measures for brucellosis transmission in Zhejiang Province include surveillance and investigation, quarantine supervision, transportation management, positive sample handling and behavioral intervention. We specifically examined the influence of the following parameters on brucellosis transmission: the proportion of live sheep transferred from Region I to Zhejiang Province ($${\theta _1}$$), the import rate of mutton from Region I to Zhejiang Province ($${\theta _2}$$), the positive detection rate of live sheep transferred from Region I ($${\rm{\sigma }}$$), the culling efficiency of infected sheep ($${k_i}$$), the proportion of contaminated mutton from Region I ($${\varepsilon _i}$$), where $${\varepsilon _i} = 0$$ with the infected sheep $${I_{si}} = 0$$, the effective infection rate from infected sheep to susceptible humans ($${\beta _{shi}}$$) and the effective infection rate from mutton to susceptible humans ($${\beta _{mhi}}$$). To better characterize the spatio-temporal heterogeneity, time-varying parameters are adopted for $${\theta _1}\left( t \right)$$, $${\theta _2}\left( t \right)$$, $${\beta _{shi}}\left( t \right)$$, and $${\beta _{mhi}}\left( t \right)$$. These parameters not only mirror the actual epidemic situation but also reflect the enforcement strength and effectiveness of prevention and control measures. The biological interpretations and units of all parameters are detailed in Table 3.

**Table 3:** Table of parameters and their descriptions

Parameters	Comments	Unit	Values	References
${{\bf{\it{A}}}_1}\left( {\bf{\it{t}}} \right)$	Number of recruitments in the sheep in Region I	head/year	Figure 9 and Table 4	Estimated
${{\bf{\it{B}}}_1}\left( {\bf{\it{t}}} \right)$	Number of births in the human in Region I	Individual/year	Figure 4 and Table 4	Interpolation
${{\bf{\it{A}}}_2}\left( {\bf{\it{t}}} \right)$	Number of recruitments in the sheep in Region II	head/year	Figure 9 and Table 4	Estimated
${{\bf{\it{B}}}_2}\left( {\bf{\it{t}}} \right)$	Number of births in the human in Region II	Individual/year	Figure 4 and Table 4	Interpolation
${{\bf{\it{\beta }}}_{{\bf{\it{ss}}}1}}\left( {\bf{\it{t}}} \right)$	Effective infection rate among infectious sheep to sheep in Region I	1/(head. year)	Figure 4 and Table 4	Estimated
${{\bf{\it{\beta }}}_{{\bf{\it{ss}}}2}}\left( {\bf{\it{t}}} \right)$	Effective infection rate among infectious sheep to sheep in Region II	1/(head. year)	Figure 9 and Table 4	Estimated
${{\bf{\it{\beta }}}_{{\bf{\it{sh}}}1}}\left( {\bf{\it{t}}} \right)$	Effective infection rate among infectious sheep to susceptible human in Region I	1/(individual. year)	Figure 9 and Table 4	Estimated
${{\bf{\it{\beta }}}_{{\bf{\it{sh}}}2}}\left( {\bf{\it{t}}} \right)$	Effective infection rate among infectious sheep to susceptible human in Region II	1/(individual. year)	Figure 9 and Table 4	Estimated
${{\bf{\it{\beta }}}_{{\bf{\it{mh}}}1}}\left( {\bf{\it{t}}} \right)$	Effective infection rate among infectious mutton to susceptible human in Region I	1/(individual. year)	Figure 9 and Table 4	Estimated
${{\bf{\it{\beta }}}_{{\bf{\it{mh}}}2}}\left( {\bf{\it{t}}} \right)$	Effective infection rate among infectious mutton to susceptible human in Region II	1/(individual. year)	Figure 9 and Table 4	Estimated
${{\bf{\it{\rho }}}_1}\left( {\bf{\it{t}}} \right)$	Sheep slaughter rate in Region I	1/year	Figure 4 and Table 4	Interpolation
${{\bf{\it{\rho }}}_2}\left( {\bf{\it{t}}} \right)$	Sheep slaughter rate in Region II	1/year	Figure 4 and Table 4	Interpolation
${{\bf{\it{k}}}_1}$	Culling rate of infected sheep in Region I	1/year	0.0400	Estimated
${{\bf{\it{k}}}_2}$	Culling rate of infected sheep in Region II	1/year	0.3951	Estimated
${\bf{\it{\mu }}}$	Average yield of mutton per sheep	kg/head	15.47	Data
${{\bf{\it{\varepsilon }}}_1}$	Proportion of contaminated mutton in Region I	none	$5.0024\times 10^{-5}$	Estimated
${{\bf{\it{\varepsilon }}}_2}$	Proportion of contaminated mutton in Region II	none	$5.0155\times 10^{-5}$	Estimated
${{\bf{\it{d}}}_{{\bf{\it{s}}}1}}$	Natural mortality rate of sheep in Region I	1/year	$5.6130\times 10^{-2}$	Estimated
${{\bf{\it{d}}}_{{\bf{\it{s}}}2}}$	Natural mortality rate of sheep in Region II	1/year	$2.9870\times 10^{-3}e$	Estimated
${{\bf{\it{d}}}_{{\bf{\it{h}}}1}}\left( {\bf{\it{t}}} \right)$	The natural mortality in the human in Region I	1/year	Figure 4 and Table 4	Interpolation
${{\bf{\it{d}}}_{{\bf{\it{h}}}2}}\left( {\bf{\it{t}}} \right)$	The natural mortality in the human in Region II	1/year	Figure 4 And Table 4	Interpolation
${{\bf{\it{\theta }}}_1}\left( {\bf{\it{t}}} \right)$	Proportion of live sheep transferred from Region I to II	none	Figure 9 and Table 4	Estimated
${{\bf{\it{\theta }}}_2}\left( {\bf{\it{t}}} \right)$	Mutton transfer rate from Region I to II	1/year	Figure 9 and Table 4	Estimated
${{\bf{\it{\theta }}}_{12}}$	Human inflow rate from Region I to II	1/year	$5.0126\times 10^{-3}$	Estimated
${{\bf{\it{\theta }}}_{21}}$	Human outflow rate from Region II	1/year	$9.4706\times 10^{-2}$	Estimated
${{\bf{\it{\delta }}}_1}$	Mutton consumption rate in Region I	1/year	0.2000	Estimated
${{\bf{\it{\delta }}}_2}$	Mutton consumption rate in Region II	1/year	0.4038	Estimated
${\bf{\it{\gamma }}}$	Recovery rate of infected human	1/(individual. year)	0.2855	Estimated
${\bf{\sigma }}$	Positive detection rate of live sheep transferred from Region I to II	none	0.8339	Estimated

^1^Estimated: Values are estimated using the least squares method, based on the reported data on human brucellosis cases in two regions provided in Subsection: Data resource 2.1, as detailed in Subsection: Parameter estimation 3.1.

^2^Interpolation: Values are given using the cubic B-spline interpolation, based on data provided in Subsection: Data resource 2.1, as detailed in Subsection: Parameter estimation 3.1.

^3^Data: Values are given based on official website, as detailed in Subsection: Data resource 2.1.

**Fig. 2:**
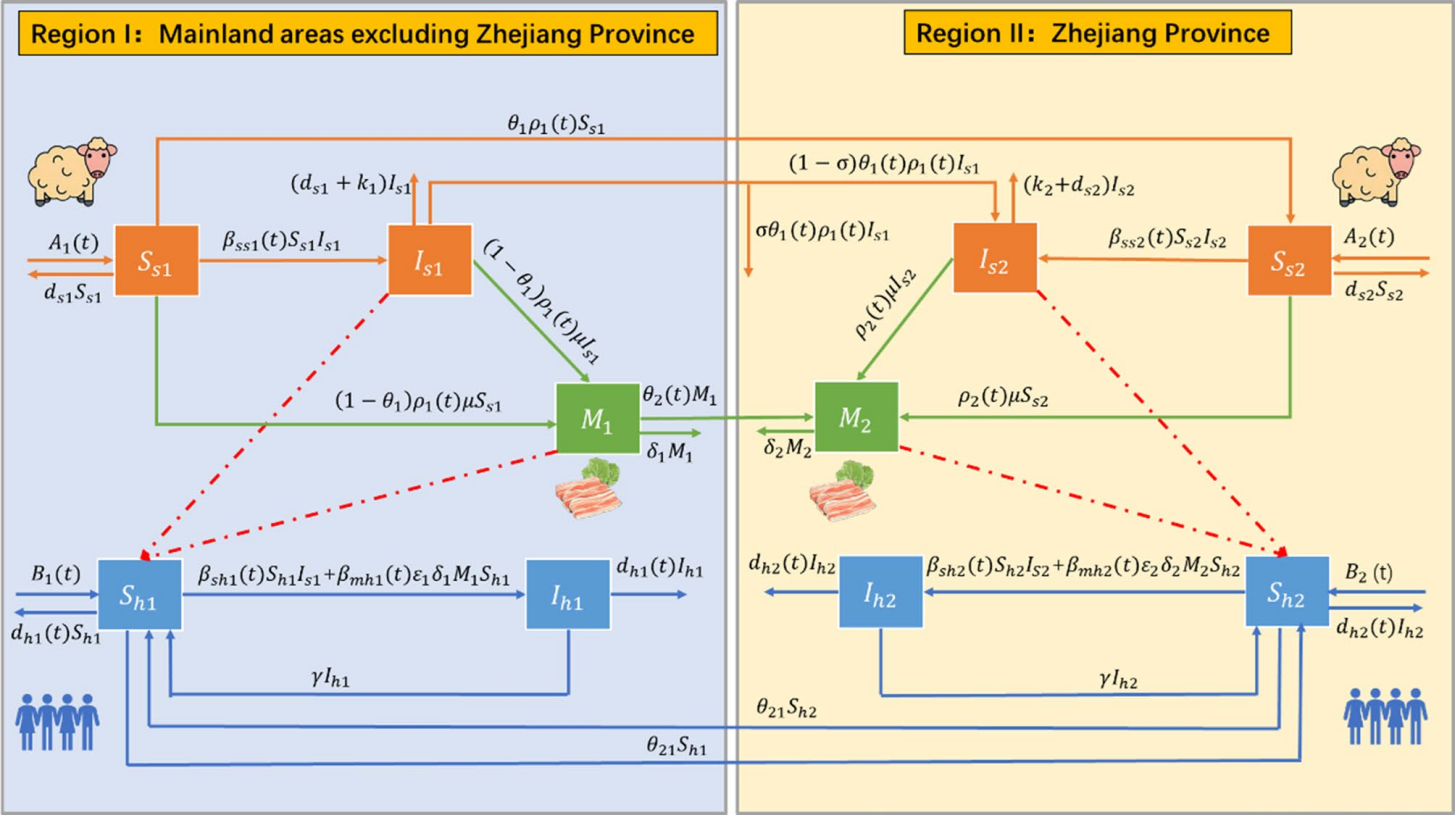
Transmission diagram of brucellosis across sheep populations, human populations, and mutton between two study regions: region I (mainland areas excluding Zhejiang Province) and Region II (Zhejiang Province). Dashed lines represent pathogen transmission routes (i.e., from sheep or mutton products to humans); solid lines denote both the state transitions of research subjects and the transportation directions of sheep/mutton, as well as intraspecific brucellosis transmission among sheep populations

**Fig. 3:**
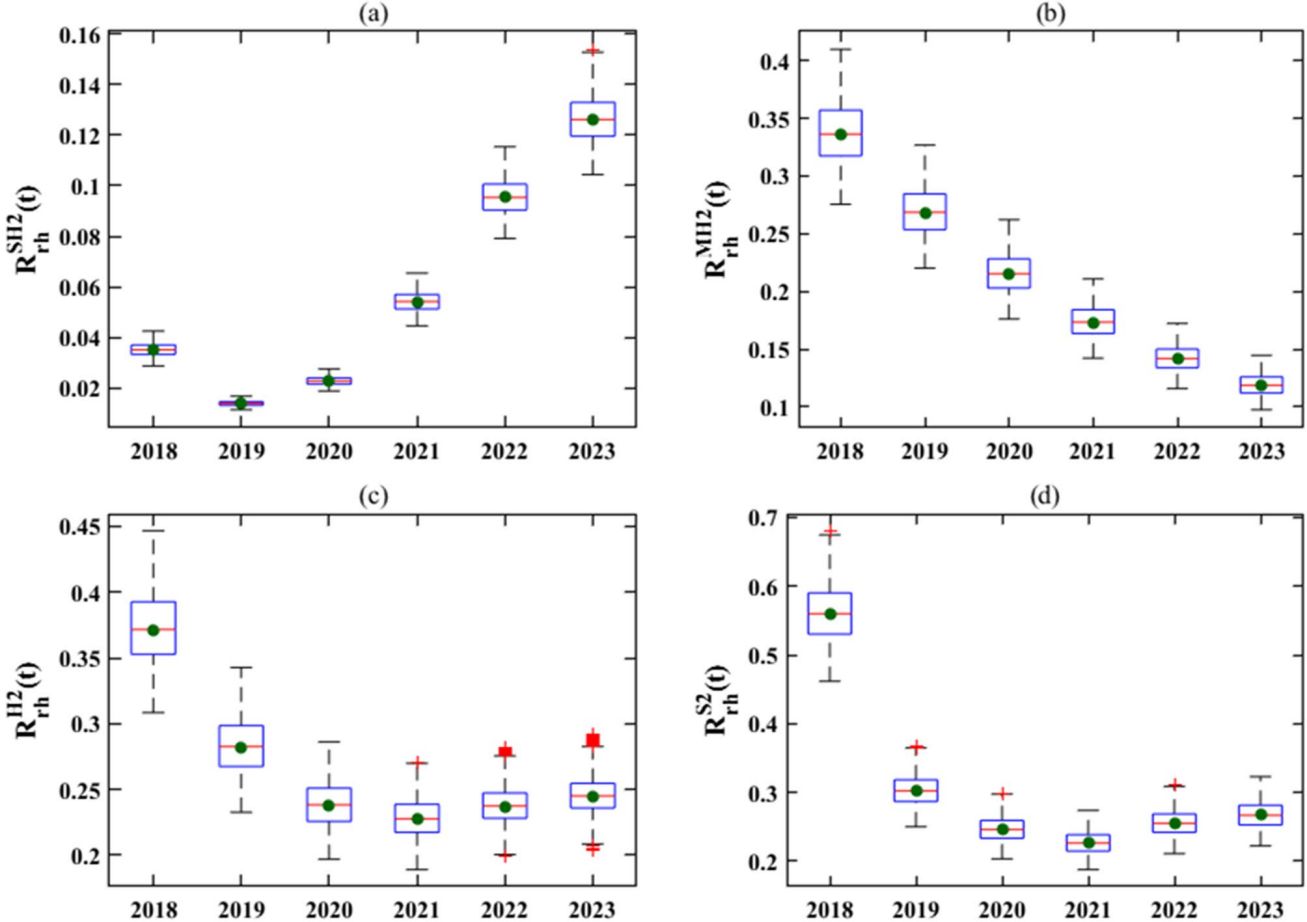
Dynamic model boxplots of the real-time reproduction numbers $R_{rt}^{SH2}\left( t \right)$, $R_{rt}^{MH2}\left( t \right), R_{rt}^{H2}\left( t \right)$ and $R_{rt}^{S2}\left( t \right)$ in Zhejiang Province (2018–2023). Simulations were performed in MATLAB R2021b with annual sampling intervals from 2018 to 2023. The results were derived from Monte Carlo random sampling, with 10,000 parameter perturbations performed within the 80–120% confidence interval of the initial variable values and parameters specified in Tables 2 and 3, followed by dynamic model simulations

**Fig. 4:**
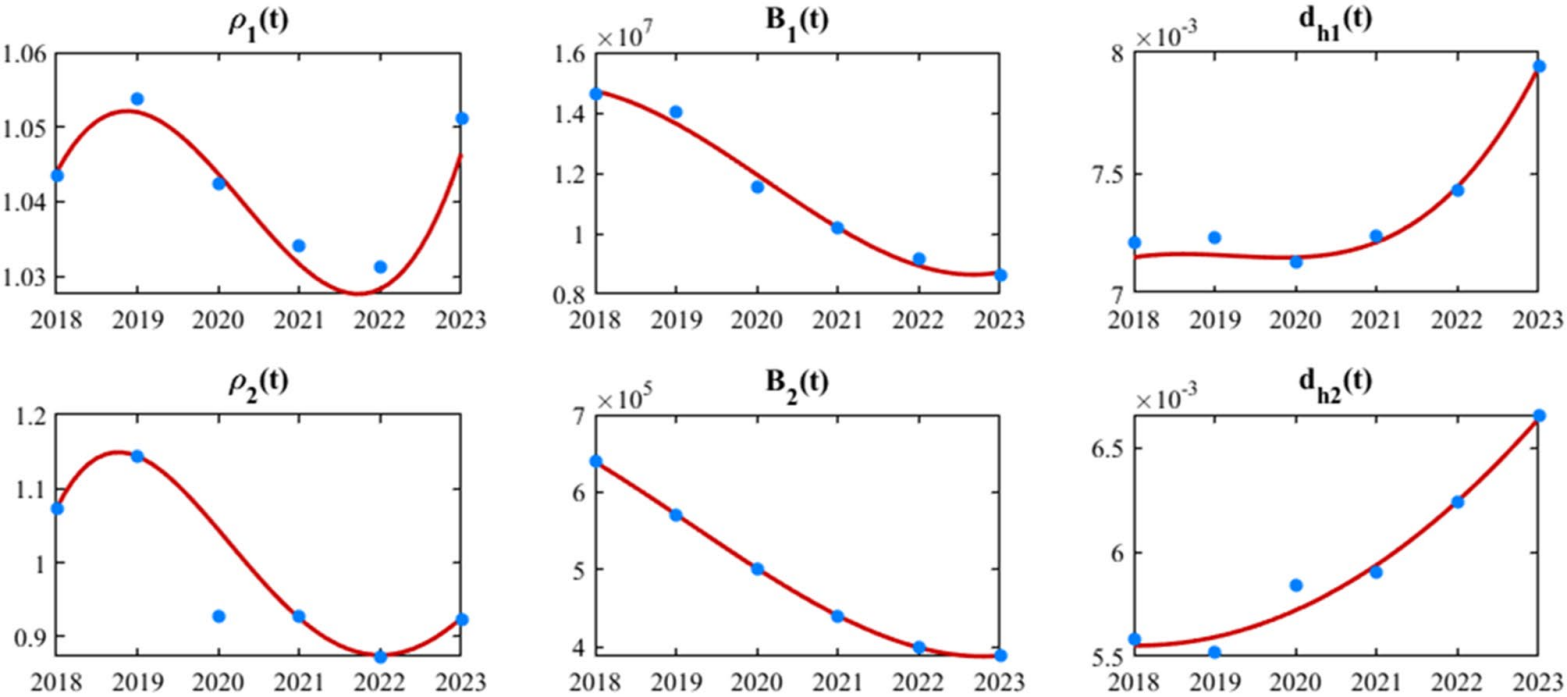
The fitting results for ${\rho _1}\left( t \right)$ and ${\rho _2}\left( t \right)$ (annual sheep slaughter rates), ${B_1}\left( t \right)$ and ${B_2}\left( t \right)$ (population birth numbers), and ${d_{h1}}\left( t \right)$ and ${d_{h2}}\left( t \right)$ (population mortality rates) in Region I and Region II from 2018 to 2023. The blue points represent the actual data, while the curves depict the fitting results

Based on the framework depicted in Fig. 2, we formulate the following non-autonomous patch dynamic model (1). The specific definitions and values of the variables and parameters included in this model are summarized in Tables 2 and 3.

1 $$\left\{ {\eqalign{&{{{d{S_{s1}}} \over {dt}} = {A_1}(t) - {\beta _{ss1}}(t){S_{s1}}{I_{s1}} - {\rho _1}(t){S_{s1}} - {d_{s1}}{S_{s1},}} & {(1.1)} \cr &{{{d{I_{s1}}} \over {dt}} = {\beta _{ss1}}(t){S_{s1}}{I_{s1}} - {\rho _1}(t){I_{s1}} - {d_{s1}}{I_{s1}} - {k_1}{I_{s1},}} & {(1.2)} \cr &{{{dM_1} \over {dt}} = {\rho _1}(t)\mu (1 - \theta _1(t))({S_{s1}} + {I_{s1}}) - {\theta _2}(t){M_1} - {\delta _1}{M_{1},}} & {(1.3)} \cr &{{{d{S_{h1}}} \over {dt}} = {B_1}(t) + \gamma {I_{h1}} + {\theta _{21}}{S_{h2}} - {\beta _{sh1}}(t){S_{h1}}{I_{s1}} - {\beta _{mh1}}(t){\varepsilon _1}{\delta _1}{M_1}{S_{h1}} - {d_{h1}}(t){S_{h1}} - {\theta _{12}}S_{h1},} & {(1.4)} \cr &{{{d{1_{h1}}} \over {dt}} = {\beta _{sh1}}(t){S_{h1}}{I_{s1}} + {\beta _{mh1}}(t){\varepsilon _1}{\delta _1}{M_1}{S_{h1}} - {d_{h1}}(t){I_{h1}} - \gamma {I_{h1},}} & {(1.5)} \cr &{{{d{S_{s2}}} \over {dt}} = {A_2}(t) + {\theta _1}(t){\rho _1}(t){S_{s1}} - {\beta _{ss2}}(t){S_{s2}}{I_{s2}} - {d_{s2}}{S_{s2}} - {\rho _2}(t){S_{s2},}} & {(1.6)} \cr &{{{d{I_{s2}}} \over {dt}} = {\beta _{ss2}}(t){S_{s2}}{I_{s2}} + (1 - \sigma ){\theta _1}(t){\rho _1}(t){I_{s1}} - {\rho _2}(t){I_{s2}} - {d_{s2}}{I_{s2}} - {k_2}{I_{s2},}} & {(1.7)} \cr &{{{d{M_2}} \over {dt}} = {\rho _1}(t)\mu ({S_{s2}} + {I_{s2}}) + {\theta _2}(t){M_1} - {\delta _2}{M_{2},}} & {(1.8)} \cr &{{{d{S_{h2}}} \over {dt}} = {B_2}(t) + \gamma {I_{h2}} + {\theta _{12}}{S_{h1}} - {\beta _{sh2}}(t){S_{h2}}{I_{s2}} - {\beta _{mh2}}(t){\varepsilon _2}{\delta _2}{M_2}{S_{h2}} - {d_{h2}}(t){S_{h2}} - {\theta _{21}}{S_{h2},}} & {(1.9)} \cr &{{{d{I_{h2}}} \over {dt}} = {\beta _{sh2}}(t){S_{h2}}{I_{s2}} + {\beta _{mh2}}(t){\varepsilon _2}{\delta _2}{M_2}{S_{h2}} - {d_{h2}}(t){I_{h2}} - \gamma {I_{h2 \cdot }}} & {(1.10)} \cr } } \right.$$

Subsequently, we will develop quantitative indicators for disease transmission using this model, with a focus on establishing important core parameters, including the infectivity rate and real-time reproduction number for interspecies transmission pathways: sheep - to - sheep, sheep - to - human, and mutton - to - human.

### Infection risk indicators

The basic reproduction number, denoted by $$ {R_0}$$, serves as a key indicator in epidemiology. It is formally defined as “the average number of secondary infections caused by an infected individual throughout their entire infectious period within a fully susceptible population” [52]. In this study, when all parameters in model (1) are treated as constants, the next-generation matrix method, initially introduced by van Driessche and Watmough [53], can be employed to derive the $${R_0}$$ specific to the model at the free-disease equilibrium. The explicit formula for $${R_0} $$ is expressed as:

2 $$\matrix{ {{R_0} = \max \left\{ {{{{\beta _{ss1}}S_{s1}^*} \over {{\rho _1} + {d_{s1}} + {k_1}}},{{{\beta _{ss2}}S_{s2}^*} \over {{\rho _2} + {d_{s2}} + {k_2}}}} \right\},} \cr } $$

where $$S_{s1}^* = {{{A_1}} \over {{\rho _1} + {d_{s1}}}}$$ and $$S_{s2}^* = {{{A_2} + {\theta _1}{\rho _1}S_{s1}^*} \over {{\rho _2} + {d_{s2}}}}$$.

In the field of epidemiology, ${R_0} < 1$ means that brucellosis will fade away without causing a widespread outbreak, while ${R_0} > 1$ indicates that brucellosis will persist following an outbreak. Building on the formulation in (2), similar analyses exploring the effects of transportation and additional factors on sheep dynamics within each patch and between patches are presented in the study with only two patches [54]. However, this indicator derived from the model with constant coefficients is mainly used in the early stages of the epidemic to assess the prevalence of brucellosis in sheep. It has limitations in reflecting the real-time dynamics of disease transmission and is difficult to quantify the specific impact of infected sheep and mutton on human health.

To more precisely evaluate the instantaneous transmission potential of brucellosis in Zhejiang Province across sheep-to-sheep, sheep-to-human, and mutton-to-human pathways, we consulted pertinent literature [54], and formulated the concept of the real-time reproduction number. Specifically, for transmission dynamics within sheep populations, the real-time reproduction number $R_{rt}^{S2}\left( t \right)$ is calculated as:

3 $$\matrix{ {R_{rt}^{S2}\left( t \right) = {\beta _{ss2}} \times {S_{s2}}\left( t \right) \times {1 \over {{d_{s2}} + {\rho _2} + {k_2}}}.} \cr } $$

This formula quantifies the average number of susceptible sheep that one infected sheep can transmit the infection to over the duration of its infectious period at time t.

For humans in Zhejiang Province, we differentiate between two distinct transmission routes: infection by contact with infected sheep and consumption of contaminated mutton. Consequently, the real-time reproduction numbers $R_{rt}^{SH2}\left( t \right)$ and $R_{rt}^{MH2}\left( t \right)$ are computed as outlined below:

4 $$\matrix{ {R_{rt}^{SH2}\left( t \right) = {\beta _{sh2}} \times {S_{h2}}\left( t \right) \times {1 \over {{d_{s2}} + {\rho _2} + {k_2}}},} \cr } $$

and

5 $$\matrix{ {R_{rt}^{MH2}\left( t \right) = {\beta _{mh2}} \times {S_{h2}}\left( t \right) \times {1 \over {{\delta _2}}}.} \cr } $$

$R_{rt}^{SH2}\left( t \right)$ denotes the average number of susceptible people that a single infected sheep can infect during its infection period at time t. Similarly, $R_{rt}^{MH2}\left( t \right)$ represents the average number of susceptible people that one kilogram of contaminated mutton can infect during the consumed period at time t. Furthermore, $R_{rt}^{H2}\left( t \right)$ signifies the real-time reproduction number for human brucellosis infections, encapsulating the combined transmission dynamics from both sheep and mutton sources. Then,

6 $$\matrix{ {R_{rt}^{H2}\left( t \right) = R_{rt}^{SH2}\left( t \right) + R_{rt}^{MH2}\left( t \right).} \cr } $$

The real-time reproduction number ${R_{rt}}\left( t \right)$ serves as a dual-purpose metric: it not only reflects the actual efficacy of epidemic control measures targeting susceptible human and sheep populations but also quantifies the potential secondary human infections stemming from each infectious source, including infected sheep and contaminated mutton. To compute this metric, we adopted a Monte Carlo random sampling approach: 10,000 parameter perturbations were executed within the 80–120% confidence interval of the baseline variables and parameters (Tables 2 and 3), with subsequent dynamic model simulations yielding the final results. This analytical framework provides a scientific basis for assessing the epidemic’s real-time transmission dynamics. As illustrated in Fig. 3, both indicators $R_{rt}^{S2}\left( t \right)$ (sheep-to-sheep) and $R_{rt}^{H2}\left( t \right)$ (overall human) exhibited a significant downward trend from 2018 to 2021 but slightly increased from 2021 to 2023. This trend underscores the success of current control measures while highlighting the need for sustained enhancement. However, the long-term efficacy of these interventions necessitates further rigorous evaluation. Further analysis shows distinct trends in transmission pathways: the sheep-to-human reproduction number $R_{rt}^{SH2}\left( t \right)$ demonstrates an upward trend, while the mutton-to-human reproduction number $R_{rt}^{MH2}\left( t \right)$ exhibits a year-over-year decline. Notably, by 2023, the mutton-to-human reproduction number has fallen below the sheep-to-human value, underscoring the escalating role of live sheep as a primary driver of human brucellosis transmission.

**Fig. 5:**
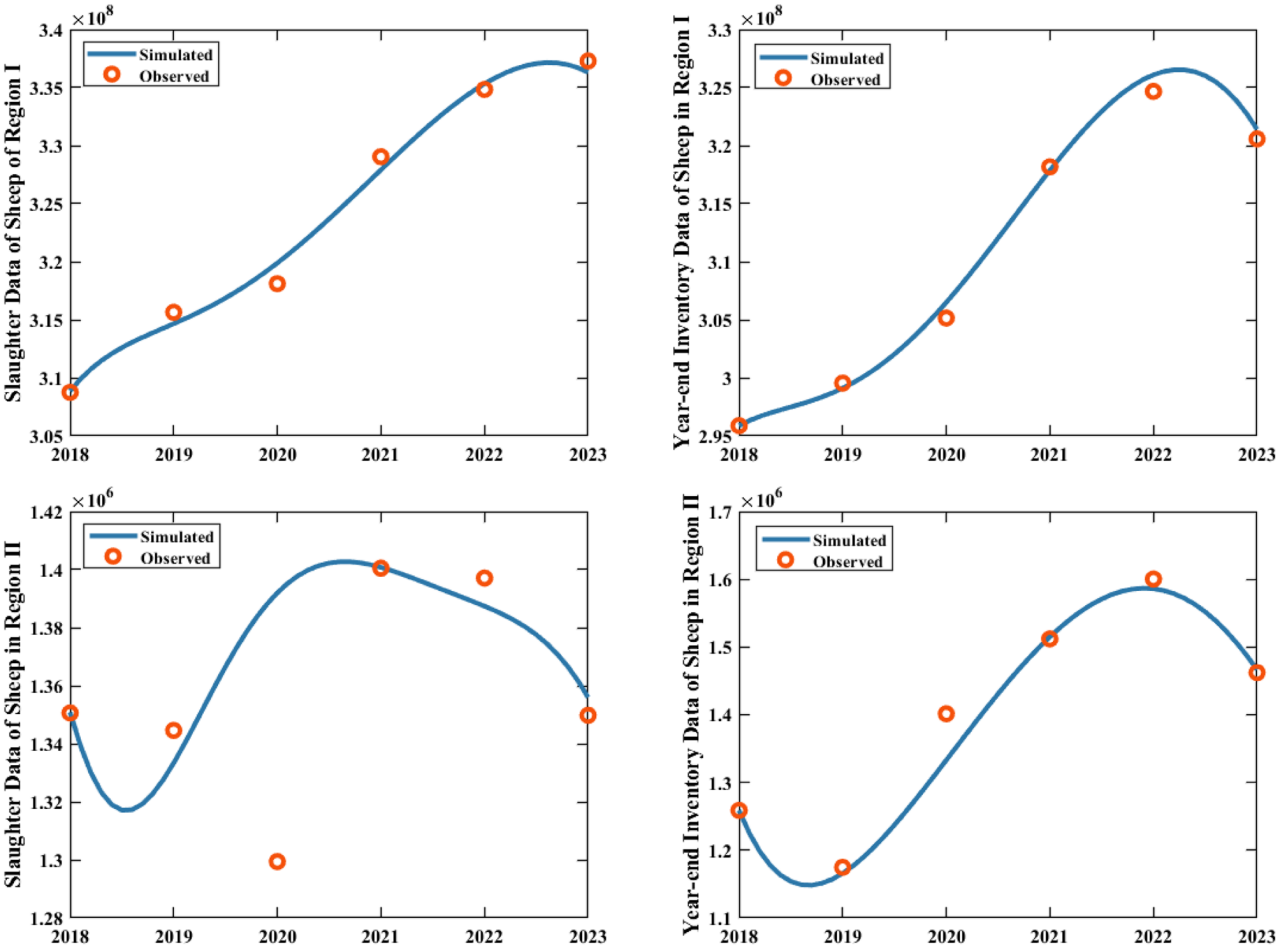
Fitting results for the year-end inventory and slaughter data of sheep in Region I and Region II from 2018 to 2023. The red dots represent the actual values, and the curves depict the fitting results

## Results

In this section, we first determine the initial variable values for the model based on the actual data obtained and referenced literature. For time-series parameters (the annual sheep slaughter rates (${\rho _1}\left( t \right)$ and ${\rho _2}\left( t \right))$, population births (${B_1}\left( t \right)$ and ${B_2}\left( t \right)$), human mortality rates (${d_{h1}}\left( t \right)$ and ${d_{h2}}\left( t \right)$)), cubic B-spline interpolation [55] is employed for nonparametric modeling to mathematically characterize them (Fig. 4). This smoothing process can effectively reduce the overfitting degree of the model to the temporal fluctuation characteristics of the raw data. Subsequently, the least squares estimation method is adopted for parameter estimation, which is implemented using the *fminsearch* command in Optimization Toolbox of MATLAB [56]. Let $\tilde X\left( t \right)$ represent the actual data (e.g., human brucellosis incidence, sheep individual level positive rate) and $X\left( t \right)$ denote the theoretical results of the model; the core objective of this estimation method is to find parameter values that minimize the value of the objective function $f\left( {h,n} \right) = \mathop \sum \limits_{i = 1}^n {\left( {X\left( t \right) - \tilde X\left( t \right)} \right)^2}$, where $h$ is the parameter vector and $n$ is the number of actual data points. The remaining unknown parameters were estimated through nonlinear fitting between the model and measured data (Fig. 9 and Table 3).

To further address the potential over-parameterization risk induced by multiple time-varying parameters in the model, a multi-layered robustness enhancement framework is constructed to ensure the reliability of the parameter estimation results: all parameter estimates are strictly constrained within biologically plausible ranges with reference to published epidemiological research findings on brucellosis transmission dynamics; specifically, the transmission rate, culling rate, proportion of infected mutton, natural mortality rate of sheep, human mortality rate, transfer rate of sheep and mutton from Region I to Region II, inflow and outflow rates of humans from Region I, mutton consumption rate, recovery rate of infected individuals, and positive detection rate are all constrained within the range of [0, 1]. An independent validation analysis is conducted using the 2024 dataset, with all model predictions falling within the 95% confidence interval, verifying the extrapolation and generalization capabilities of the proposed model. To identify the key drivers of the epidemic transmission, we conduct sensitivity analysis using Partial Rank Correlation Coefficients (PRCCs) [57] to quantitatively evaluate the contribution of parameters to the temporal variations in human brucellosis incidence and sheep individual level positive rate in Zhejiang Province. The results of the global sensitivity analysis further indicate that the model outputs are mainly driven by a small subset of key parameters, suggesting that the model structure does not overly depend on non-critical parameters. These comprehensive measures synergistically guarantee the stability of the parameter estimation results and their epidemiological rationality, thereby mitigating the inherent over-parameterization risk of the model based on limited 6-year observational data.

Finally, based on the established model, we perform multi-scenario simulation predictions for the epidemic trends of brucellosis in Zhejiang Province from 2024 to 2026. Specifically, the analyses includ: 1) Quantitative assessment of dominant factors: Gradient simulations are given for key parameters such as live sheep import volume, stocking scale, and mutton import volume; 2) Verification of prevention and control efficacy: A systematic evaluation of the inhibitory effects of prevention and control parameters (e.g., positive sheep in breeding/transportation links and positive mutton in consumption links) on epidemic progression; 3) Optimization of prevention and control strategies: Exploration of synergistic effects of multi-dimensional interventions through combined simulations, including comprehensive detection systems (“breeding + transportation + consumption” three-link monitoring), transportation mode transformation (shift from “live animal transportation” to “meat transportation”), and changes in protective awareness (direct contact rate).

### Parameter estimation

First, referring to the year-end sheep inventory of Region I in 2018, we estimated the initial values for susceptible sheep, ${S_{s1}}\left( 0 \right) = 2.9288 \times {10^8}$, and the infected sheep, ${I_{s1}}\left( 0 \right) =$4.9898$\times {10^6}$. Referencing the year-end sheep inventory and combining the sheep positivity rate of 0.09% in Region II at that time [58], we estimated the initial values for susceptible sheep, ${S_{s2}}\left( 0 \right) = 1.2577 \times {10^6}$, and infected sheep, ${I_{s2}}\left( 0 \right) =$1133. From the population data from 2018 to 2023 obtained from the National Bureau of Statistics of China [43] and the Zhejiang Bureau of Statistics [44], we derived ${S_{h1}}\left( 0 \right) = 1.3327 \times {10^9}$ and ${S_{h2}}\left( 0 \right) = 6.2730 \times {10^7}$. Subsequently, to align with the model’s initial conditions and reported brucellosis cases, we estimated ${S_{h1}}\left( 0 \right) = 1.3400 \times {10^9}$. Based on the annual epidemic data of the National Disease Control and Prevention Administration from 2018 to 2023 [37] and the corresponding data from the Health Commission of Zhejiang Province [38], ${I_{h1}}\left( 0 \right) = 5.9625 \times {10^4}$ and ${I_{h2}}\left( 0 \right) =$158.

Second, using the collected annual sheep slaughter quantity, sheep inventory, population births, and population deaths in Region I (representing all areas except Zhejiang Province) and Region II (specifically Zhejiang Province), we calculated the annual sheep slaughter rate (slaughter quantity/inventory) and human mortality rate (population deaths/permanent population). We then employed cubic B-spline interpolation to perform fitting analysis between the monitoring data and the actual conditions, where detailed methodological descriptions are provided in Appendix A.2. Through this process, we obtained key parameters, including ${\rho _1}\left( t \right)$, ${\rho _2}\left( t \right)$, ${B_1}\left( t \right)$, ${B_2}\left( t \right)$), ${d_{h1}}\left( t \right)$ and ${d_{h2}}\left( t \right)$ for Regions I and II. The fitting results are presented in Fig. 4, where the high degree of consistency between the fitted curves and real data is visually evident.

Next, we define ${N_{s1}}\left( t \right)$ and ${N_{s2}}\left( t \right)$ as the year-end sheep inventory for Region I and Region II, respectively, where ${N_{s1}}\left( t \right) = {S_{s1}}\left( t \right) + {I_{s1}}\left( t \right)$ and ${N_{s2}}\left( t \right) = {S_{s2}}\left( t \right) + {I_{s2}}\left( t \right)$. Based on the first two equations (1.1) + (1.2) and equations (1.6) + (1.7) in the constructed dynamic model (1), we derived differential equations (7) and (8) describing the temporal changes in the year-end sheep inventory for the two regions:

7 $$\matrix{ {{{d{N_{s1}}} \over {dt}} = {A_1}\left( t \right) - {\rho _1}\left( t \right){N_{s1}} - {d_{s1}}{N_{s1}},} \cr } $$

8 $$\matrix{ {{{d{N_{s2}}} \over {dt}} = {A_2}\left( t \right) + {\theta _1}\left( t \right){\rho _1}\left( t \right){N_{s1}} - {\rho _2}\left( t \right){N_{s2}} - {d_{s2}}{N_{s2}}.} \cr } $$

To improve the accuracy of parameter estimation, we utilized the year-end sheep inventory and annual sheep slaughter data for Region I and Region II from 2018 to 2023. Through the least squares approach, we simultaneously fitted the above equations (7) and (8) to the corresponding regional year-end inventory and slaughter data, estimating the annual sheep additions (${A_1}\left( t \right)$ and ${A_2}\left( t \right)$) and natural mortality rates of sheep rates (${d_{s1}}$ and ${d_{s2}}$) for two regions. The fitting results for the year-end sheep inventory and slaughter are shown in Fig. 5. The estimated values of ${d_{s1}}$ and ${d_{s2}}$ are presented in Table 3, while the estimated curves and corresponding numerical expressions for ${A_1}\left( t \right)$ and ${A_2}\left( t \right)$ are provided in Fig. 9 and Table 4, respectively.

**Fig. 6:**
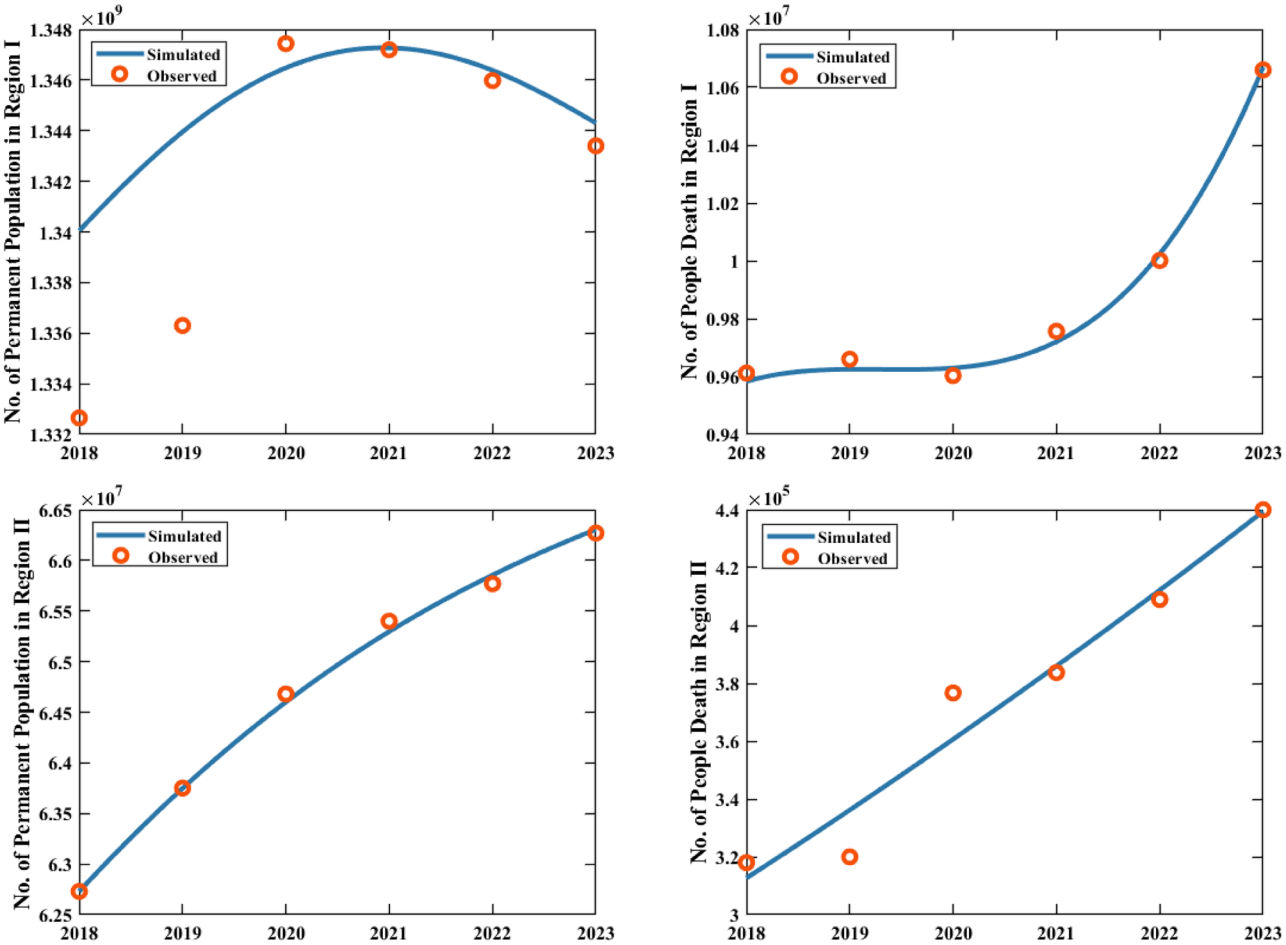
Fitting results for the permanent population and death numbers of two regions from 2018 to 2023. The red dots represent the actual values, and the curves depict the fitting results. The higher population size in Region I in 2020 compared to 2019 is due to the seventh national population census conducted in 2020

**Table 4:** Time-varying coefficient parameter expression

Parameters	Expressions
${A_1}\left( t \right)$	$- 1.3178 \times {{10}^6}{t^3} +1.0985 \times {{10}^7}{t^2} - 1.8381 \times {{10}^7}t + 3.3916 \times {{10}^8}$
${B_1}\left( t \right)$	$9.2430 \times {{10}^4}{t^3} - 8.6194 \times {{10}^5}{t^2} + 8.5879 \times {{10}^5}t + 1.4633 \times {{10}^7}$
${A_2}\left( t \right)$	$1.2647 \times {{10}^4}{t^3} - 2.3250 \times {{10}^5}{t^2} + 1.1734 \times {{10}^6}t - 8.2561 \times {{10}^5}$
${B_2}\left( t \right)$	$1.8296 \times {{10}^3}{t^3} - 1.2068 \times {{10}^4}{t^2} - 4.4198 \times {{10}^4}t + 6.9333 \times {{10}^5}$
${\beta _{ss1}}\left( t \right)$	$4.0000 \times {{10}^{ - 11}}{t^2} - 1.0017 \times {{10}^{ - 10}}t + 2.9988 \times {{10}^{ - 9}}$
${\beta _{sh1}}\left( t \right)$	$- 2.3501 \times {{10}^{ - 13}}{t^3} + 2.2455 \times {{10}^{ - 12}}{t^2} - 2.9600 \times {{10}^{ - 12}}t +5.9401 \times {{10}^{ - 12}}$
${\beta _{mh1}}\left( t \right)$	$- 1.7901 \times {{10}^{ - 13}}{t^3} + 1.8951 \times {{10}^{ - 12}}{t^2} - 1.9983 \times {{10}^{ - 12}}t +7.1501 \times {{10}^{ - 12}}$
${\beta _{ss2}}\left( t \right)$	$- 4.5404 \times {{10}^{ - 9}}{t^3} + 8.4793 \times {{10}^{ - 8}}{t^2} - 4.8107 \times {{10}^{ - 7}}t + 1.0561 \times {{10}^{ - 6}}$
${\beta _{sh2}}\left( t \right)$	$- 4.1119 \times {{10}^{ - 11}}{t^3} + 5.7691 \times {{10}^{ - 10}}{t^2} - 1.9310 \times {{10}^{ - 9}}t + 2.2195 \times {{10}^{ - 9}}$
${\beta _{mh2}}\left( t \right)$	$2.9504 \times {{10}^{ - 12}}{t^3} + 7.0682 \times {{10}^{ - 11}}{t^2} - 6.5637 \times {{10}^{ - 10}}t + 2.7529 \times {{10}^{ - 9}}$
${\rho _1}\left( t \right)$	$0.0021{t^3} -0.0208{t^2} + 0.0558t + 1.0068$
$${\rho _2}\left( t \right)$$	$$ - 0.0025{t^4} + 0.0500{t^3} - 0.3226{t^2} + 0.7260t + 0.6222$$
${d_{h1}}\left( t \right)$	$1.5648 \times {{10}^{ - 5}}{t^3} - 1.0413 \times {{10}^{ - 4}}{t^2} + 2.1308 \times {{10}^{ - 4}}t + 7.0270 \times {{10}^{ - 3}}$
${d_{h2}}\left( t \right)$	$4.4286 \times {{10}^{ - 5}}{t^2} - 9.2286 \times {{10}^{ - 5}}t + 5.6000 \times {{10}^{ - 3}}$
${\theta _1}\left( t \right)$	$2.3293 \times {{10}^{ - 6}}{t^3} +2.3991 \times {{10}^{ - 5}}{t^2} - 4.8302 \times {{10}^{ - 4}}t + 3.2009 \times {{10}^{ - 3}}$
${\theta _2}\left( t \right)$	$5.5279 \times {{10}^{ - 7}}{t^3} + 1.6499 \times {{10}^{ - 5}}{t^2} - 2.2012 \times {{10}^{ - 6}}t +9.8893 \times {{10}^{ - 5}}$

Note: $t$ starts at 1，where $t = \left( {1,{\rm{ }}2,{\rm{ }}3,{\rm{ }}4,{\rm{ }}5,{\rm{ }}6} \right)$ corresponds to the years (2018, 2019, 2020, 2021, 2022, 2023)

Next, we define ${N_{h1}}\left( t \right)$ and ${N_{h2}}\left( t \right)$ as the total permanent population in Region I and Region II, respectively, where ${N_{h1}}\left( t \right) = {S_{h1}}\left( t \right) + {I_{h1}}\left( t \right)$ and ${N_{h2}}\left( t \right) = {S_{h2}}\left( t \right) + {I_{h2}}\left( t \right)$. Based on the system of equations in the dynamic model (1), specifically equations (1.4) + (1.5) and equations (1.9) + (1.10), we derived differential equations (9) and (10) describing the temporal variations in the permanent population of the two regions:

9 $$\matrix{ {{{d{N_{h1}}} \over {dt}} = {B_1}\left( t \right) + {\theta _{21}}{S_{h2}} - {d_{h1}}\left( t \right){N_{h1}} - {\theta _{12}}{S_{h1}},} \cr } $$

10 $$\matrix{ {{{d{N_{h2}}} \over {dt}} = {B_2}\left( t \right) - {\theta _{21}}{S_{h2}} - {d_{h2}}\left( t \right){N_{h2}} + {\theta _{12}}{S_{h1}}.} \cr } $$

Using the collected data on the permanent population and deaths in the two regions, simultaneously fitted differential equations (9) and (10) to the observed data via least squares estimation. Through this process, we estimated the population inflow rate from Region I to Zhejiang Province (${\theta _{12}}\left( t \right)$), and the population outflow rate from Zhejiang Province (${\theta _{21}}\left( t \right)$). The fitting results are shown in Fig. 6. Notably, given the significant population growth revealed by the 2020 national census data, we adjusted the initial total population of Region I accordingly to optimize the fitting performance.

**Fig. 7:**
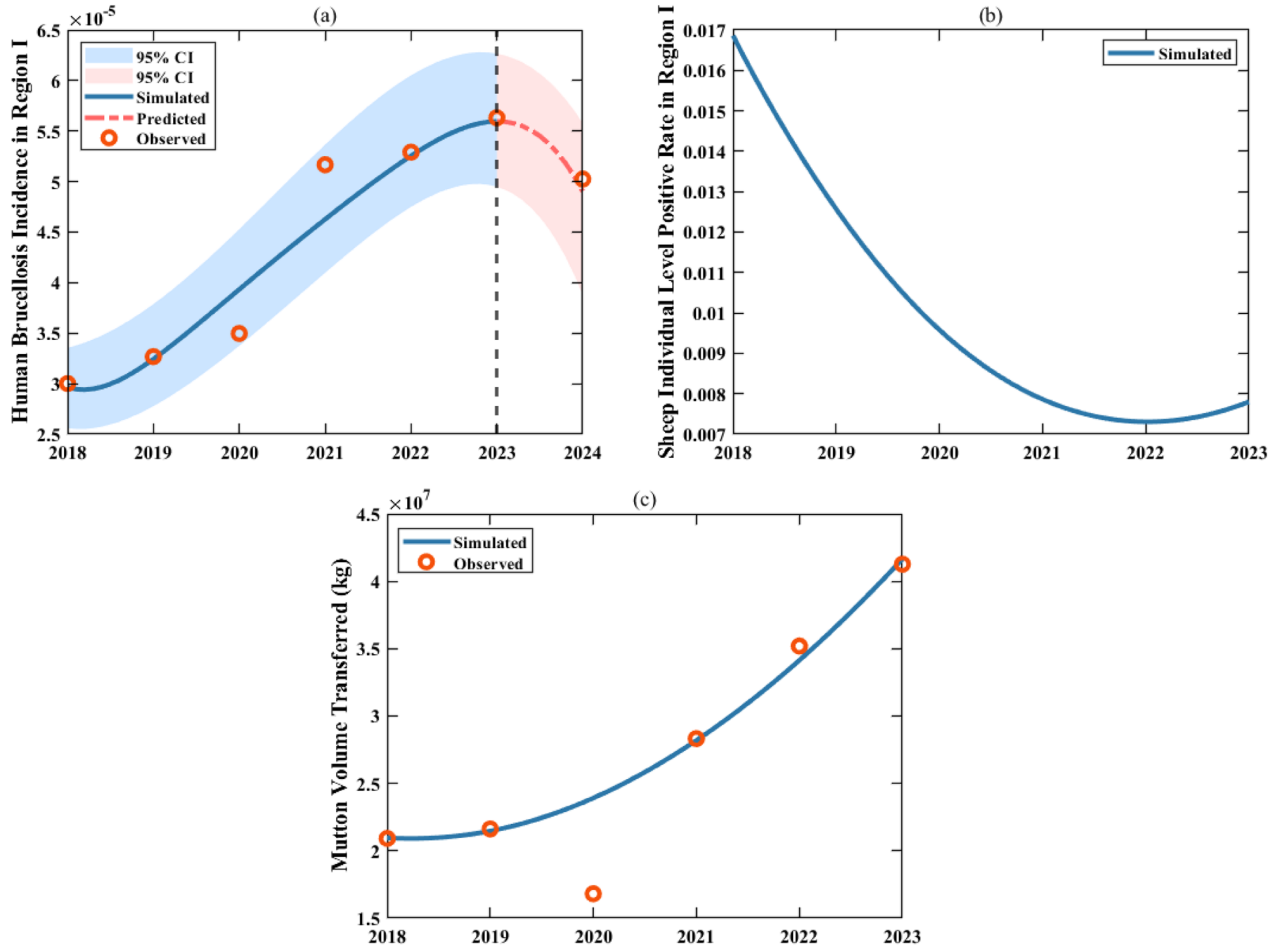
Fitting results for human brucellosis incidence, sheep individual level positive rate in Region I, and the mutton volume transferred to Zhejiang Province from 2018 to 2024. For the number of infected individuals in Region I, the solid blue line represents the fitting curve from 2018 to 2023, the red dashed line represents the 2024 case projections using the fitted parameters, the red dots represent the actual data, and the blue and pink shaded areas indicate the 95% confidence interval of the fitting results

**Fig. 8:**
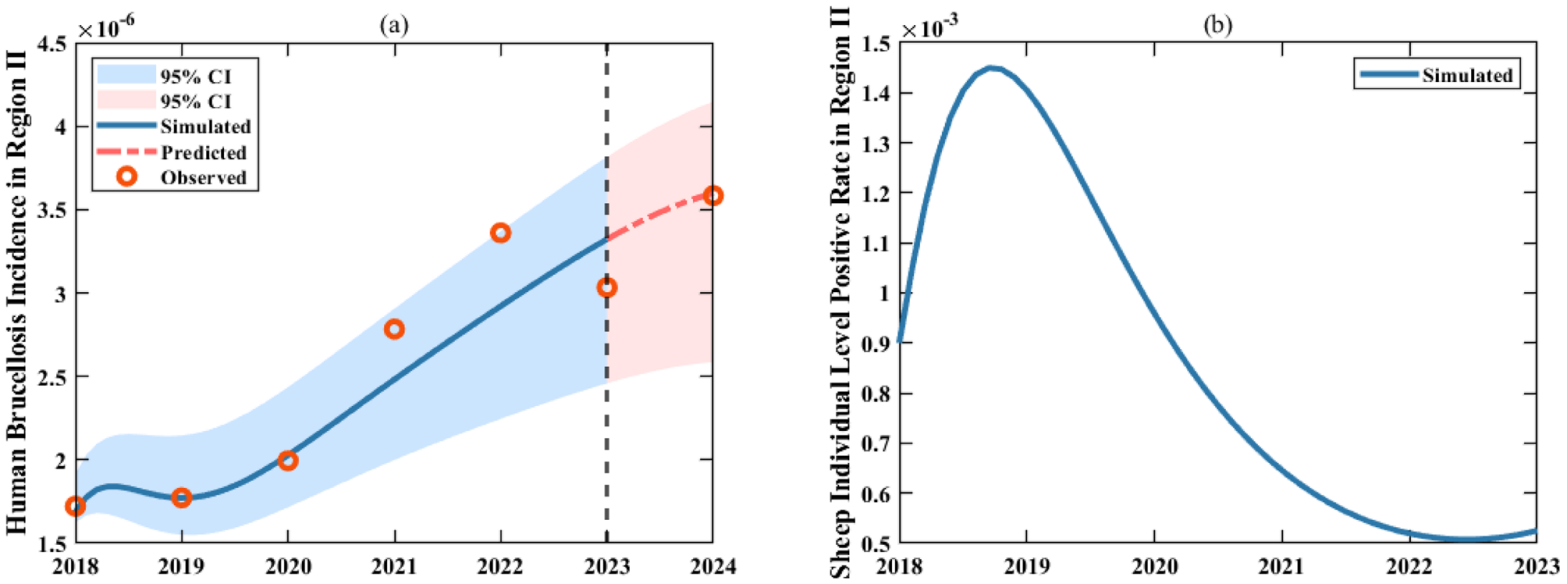
Fitting results for human brucellosis incidence and sheep individual level positive rate in Zhejiang Province from 2018 to 2024. For the human brucellosis incidence in Zhejiang Province, the solid blue line represents the fitting curve from 2018 to 2023, the red dashed line represents the 2024 case projections using the fitted parameters, the red dots represent the actual data, and the blue and pink shaded areas indicate the 95% confidence interval of the fitting results

**Fig. 9:**
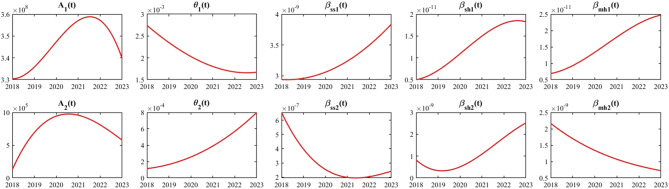
Fitting results for ${A_1}\left( t \right)$ and ${A_2}\left( t \right)$, ${\theta _1}\left( t \right)$ and ${\theta _2}\left( t \right)$, ${\beta _{ss1}}\left( t \right)$ and ${\beta _{ss2}}\left( t \right)$, ${\beta _{sh1}}\left( t \right)$ and ${\beta _{sh2}}\left( t \right)$, and ${\beta _{mh1}}\left( t \right)$ and ${\beta _{mh2}}\left( t \right)$ in Region I and Region II from 2018 to 2023

Finally, for parameters and initial variable conditions that were not explicitly determined, we adopted the following strategies. These parameters include: For Region I: the proportion of sheep transferred from Region I to Region II, (${\theta _1}\left( t \right)$), the rate of mutton transferred from Region I to Region II (${\theta _2}\left( t \right)$), the proportion of contaminated mutton, ${\varepsilon _1}$, the mutton consumption rate, ${\delta _1}$, the effective infection rate among infectious sheep to susceptible sheep, ${\beta _{ss1}}\left( t \right)$, the effective infection rate among infectious sheep to susceptible human, ${\beta _{sh1}}\left( t \right)$, the effective infection rate among contaminated mutton to susceptible human, ${\beta _{mh1}}\left( t \right)$, the culling rate of infected sheep, ${k_1}$; the initial number of infected sheep, ${I_{s1}}\left( 0 \right)$, the initial mutton quantity, ${M_1}\left( 0 \right)$; the initial number of susceptible humans, ${S_{h1}}\left( 0 \right)$. And for Region II, the proportion of contaminated mutton, ${\varepsilon _2}$, the mutton consumption rate, ${\delta _2}$, the effective infection rate among infectious sheep to susceptible sheep, ${\beta _{ss2}}\left( t \right)$, the effective infection rate among infectious sheep to susceptible sheep, ${\beta _{sh2}}\left( t \right)$, the effective infection rate among contaminated mutton to susceptible, ${\beta _{mh2}}\left( t \right)$, the culling rate of infected sheep, ${k_2}$, the initial mutton quantity, ${M_2}\left( 0 \right)$, and the positive detection rate of sheep transferred from Region I to Region II, ${\rm{\sigma }}$. To estimate these parameters, we employed the equations (1.1) - (1.5) and (1.6) - (1.10) in the model, fitting them to human brucellosis incidence data from 2018 to 2023 and concurrent data on live sheep and mutton imports. Additionally, we reserved the 2024 human brucellosis incidence data as a validation set for subsequent model evaluation. Notably, due to potential underreporting or misreporting in brucellosis surveillance, the collected data inherently contain errors. To systematically account for and quantify this uncertainty, we assumed that human brucellosis data in both regions follow a Poisson distribution, treating the mean value at each time point as the observed value [59]. During the fitting, we randomly sampled 5000 datasets from the Poisson distribution [60] and performed 5000 least squares calculations in MATLAB R2021b [61]. These computations yielded the mean values or numerical expressions for all estimated parameters, as shown in Tables 1 and 2. To visualize the fitting performance, we plotted the results based on these parameter estimates (Figs. 7, 8, and 9). From Figs. 7(a) and 8(a), it is evident that the modeled human brucellosis incidences in Region I and Zhejiang Province in 2024 closely align with the reported data, with projected values of 4.90 per 100,000 (95% CI: 4.86–4.94) and 3.60 per 100,000 (95% CI: 3.46–3.74), respectively, all falling within the 95% confidence bands of the model.

### Sensitivity analysis

**Fig. 10:**
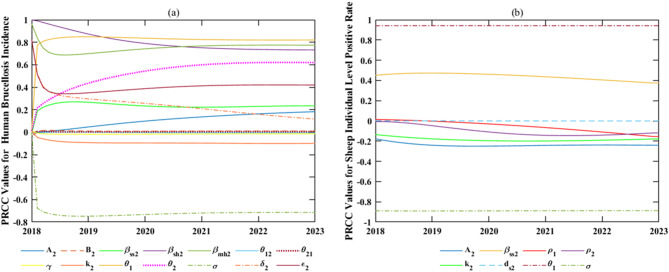
Results of the sensitivity analysis of the temporal variation in human brucellosis incidence rate and sheep individual level positive rate

We adopted Latin Hypercube Sampling (LHS) with a sample size of 5000 to evaluate the partial rank correlation coefficients (PRCCs) between input parameters and output variables (i.e., human brucellosis incidence rate and individual sheep positivity rate). This sample size ensures the numerical stability of PRCC estimates and aligns with common practices in global sensitivity analysis [62, 63]. All parameters were subjected to uniform random perturbation within the range of ±20% of their baseline estimates (equivalent to 80–120%), a range designed to capture practical uncertainties inherent in monitoring data and parameter estimation while excluding biologically implausible extremes, thus guaranteeing the robustness and interpretability of sensitivity analysis results. Due to the absence of priori distribution information and available data on input parameters, we assumed uniform distribution for all parameters: constant parameters were assigned a fluctuation range of 80% to 120% of their estimated values (Table 3). For time-varying parameters, we adopted the range from the minimum to the maximum values according to Table 4. The results of the sensitivity analysis are displayed in Fig. 10.

Figure 10(a) presents the results of a sensitivity analysis on the temporal changes in the incidence rate of human brucellosis in Zhejiang Province. Among the parameters, the contact rate of susceptible individuals with infected mutton (${\beta _{mh2}}$) and the contact rate of susceptible individuals in Zhejiang Province with infected sheep (${\beta _{sh2}}$) consistently showed a strong correlation (|PRCC|≥0.4), both of which were positive. Secondly, the |PRCC| value of the proportion of infected mutton in Zhejiang Province (${\varepsilon _2}$) exhibited a fluctuating trend of “first decreasing and then increasing”, showing a moderate positive correlation overall. The |PRCC| value of the rate of human mutton consumption (${\delta _2}$) decreased year by year from 0.8252 to 0.1165, with the correlation weakening from strong to weak and showing a positive correlation. The |PRCC| value of the effective transmission rate of infected sheep to susceptible sheep in Zhejiang Province (${\beta _{ss2}}$) demonstrated a fluctuating pattern of “increasing - decreasing”, which was always below 0.4, indicating a weak positive correlation. It is noteworthy that the correlations of the proportion of sheep transported from Region I to Zhejiang (${\theta _1}$), the rate of mutton transported from Region I to Zhejiang (${\theta _2}$), and the positive detection rate of live sheep transported into Zhejiang (${\rm{\sigma }}$) gradually increased from weak to strong, and reached a strong correlation as early as before 2019. Both ${\theta _1}$ and ${\theta _2}$ were positively correlated with the human incidence rate, while ${\rm{\sigma }}$ was negatively correlated. In addition, the number of newly added sheep in Zhejiang Province (${A_2}$), the number of newly added population in Zhejiang Province (${B_2}$), the rate of population inflow from Region I to Zhejiang (${\theta _{12}}$), the rate of population outflow from Zhejiang to Region I (${\theta _{21}}$), the recovery rate of infected individuals ($\gamma$), and the culling rate of infected sheep (${k_2}$) had consistently weak impacts on the individual positive rate (|PRCC|≤0.2).

Figure 10(b) shows the results of the sensitivity analysis on the temporal changes in the individual positive rate of sheep as affected by parameters. The results indicated that ${\theta _1}$ and ${\rm{\sigma }}$ maintained a strong correlation throughout the period (|PRCC|≥0.4), with their |PRCC| values both consistently exceeding 0.8. Specifically, ${\theta _1}$ was positively correlated, while ${\rm{\sigma }}$ was negatively correlated. ${\beta _{ss2}}$ showed a moderate positive correlation with the individual positive rate of sheep. The |PRCC| value of ${A_2}$ increased from 0.1774 to 0.2408, which was negatively correlated with the individual positive rate of sheep in Zhejiang Province. Moreover, ${k_2}$, the natural mortality and culling rate of sheep in Zhejiang Province (${d_{s2}}$), the slaughter rate of sheep in Region I (${\rho _1}$), and the slaughter rate of sheep in Zhejiang Province (${\rho _2}$) had consistently weak impacts on the individual positive rate of sheep (|PRCC|≤0.2).

### Numerical simulation

#### Quantitative assessment of dominant factor impacts

When analyzing the dominant factors influencing brucellosis transmission risk in Zhejiang Province, we examined the dynamic effects of three parameters on human brucellosis incidence, the individual - level sheep positive rate and the number of infected humans: the proportion of sheep transferred in Zhejiang Province $({\theta _1})$, the annual average number of new sheep $\left( {{A_2}} \right)$ and the rate of mutton transferred into Zhejiang Province $({\theta_2})$. In addition, this study took the epidemic situation predicted by the model in 2026 as the baseline scenario and quantitatively compared the inhibitory effects of different intervention strategies on the spread of brucellosis. The complete numerical results of all simulation scenarios are summarized in Appendix A.1.

**Fig. 11:**
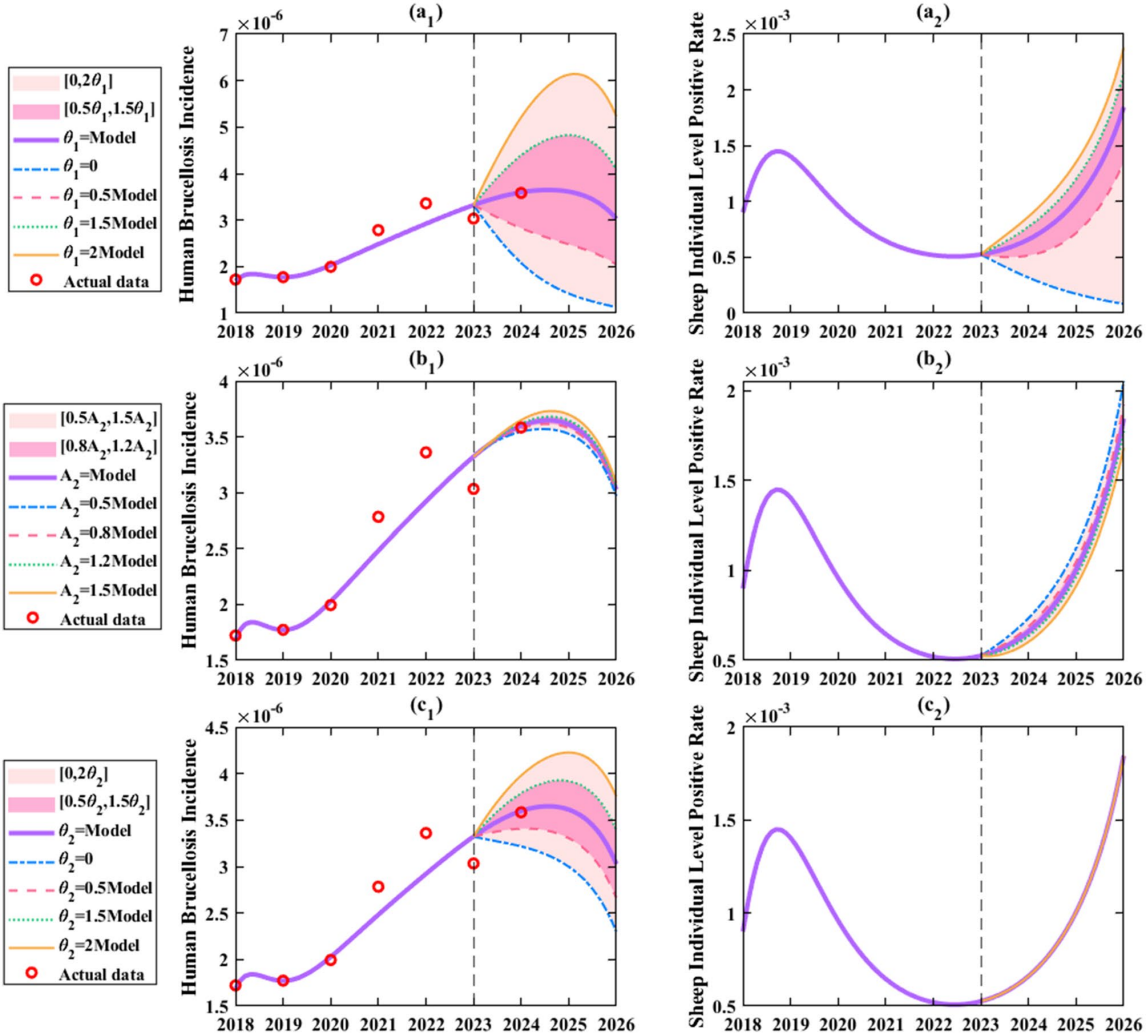
Simulation results of the assessment of human brucellosis incidence and sheep individual level positive rate in Zhejiang Province under different dominant factors: a. Increasing or decreasing the proportion of imported live sheep (${\theta _1}$); b. Increasing or decreasing annual sheep additions (${A_2}$); c. Increasing or decreasing the mutton import rate (${\theta _2}$)

As shown in Fig. 11(a_1_), under current conditions without any intervention adjustments, human brucellosis incidence would remain stably controlled below 0.4 per 100,000 and exhibit a downward trend. Under the baseline scenario with no interventions, the incidence in 2026 would remain at 0.3 per 100,000. sheep individual level positive rate would also stay below 0.2%, it is estimated that the positivity rate will be 0.18% in 2026, showing an upward trend (Fig. 11(a_2_)). Further analysis revealed that doubling ${\theta _1}$ would cause human incidence to first increase to a peak of 0.6 per 100,000 (Fig. 11(a_1_)) in 2025 before declining, resulting in a 71.4% increase relative to the no-intervention baseline. Concurrently, sheep individual level positive rate would rise to 0.25% (Fig. 11(a_2_)), resulting in a 38.9% rise above the 2026 no-intervention baseline. Conversely, implementing an extreme measure to completely cease live sheep imports would reduce human incidence to below 0.15 per 100,000 (a 67% reduction, Fig. 11 (a_1_)) and seroprevalence to below 0.02% (decreased by 91.67%, Fig. 11 (a_2_)), effectively controlling the epidemic. Fig. 11(b_1_) and (b_2_) indicated that annual sheep additions ${A_2}$ had moderate impacts on both human incidence and sheep individual level positive rate, contributing little to fluctuations in infected individuals. Fig. 11(c_1_) and (c_2_) demonstrated the effects of mutton import rate changes. Fig. 11(c_1_) shows, doubling ${\theta _2}$ would lead to a biphasic trend in human incidence (initial increase followed by decrease), with a projected rise to 0.37 per 100,000 (a 23.3% increase) and 253 infected individuals by 2026. Ceasing mutton imports entirely would reduce incidence to below 0.25 per 100,000 (decreased by 20%), and infected individuals to 155, achieving strong epidemic control. Moderate increases in ${\theta _2}$ (e.g., 1.5-fold) would raise incidence to 0.35 per 100,000 (a 16.7% increase), whereas reducing ${\theta _2}$ by half would lower incidence to 0.27 per 100,000 (decreased by 10%), with a downward trend, which is beneficial for control.

#### Evaluation of prevention and control measure efficacy

When analyzing the impacts of prevention and control measures on brucellosis transmission risk in Zhejiang Province, we investigated the specific mechanisms through which changes in multiple key links affect human brucellosis incidence, the sheep individual level positive rate, and the number of infected humans. These key factors include: breeding link detection efficiency (culling proportion of infected sheep, ${k_2}$), transportation link detection efficiency (positive detection rate of sheep imported from Region I to Zhejiang Province, ${\rm{\sigma }}$), consumption link detection efficiency (proportion of contaminated mutton, ${\varepsilon _2}$), as well as protection awareness in the breeding and consumption links (reduction in effective contact between susceptible humans and infected sheep ${\beta _{sh2}}$ or contaminated mutton ${\beta _{mh2}}$).

**Fig. 12:**
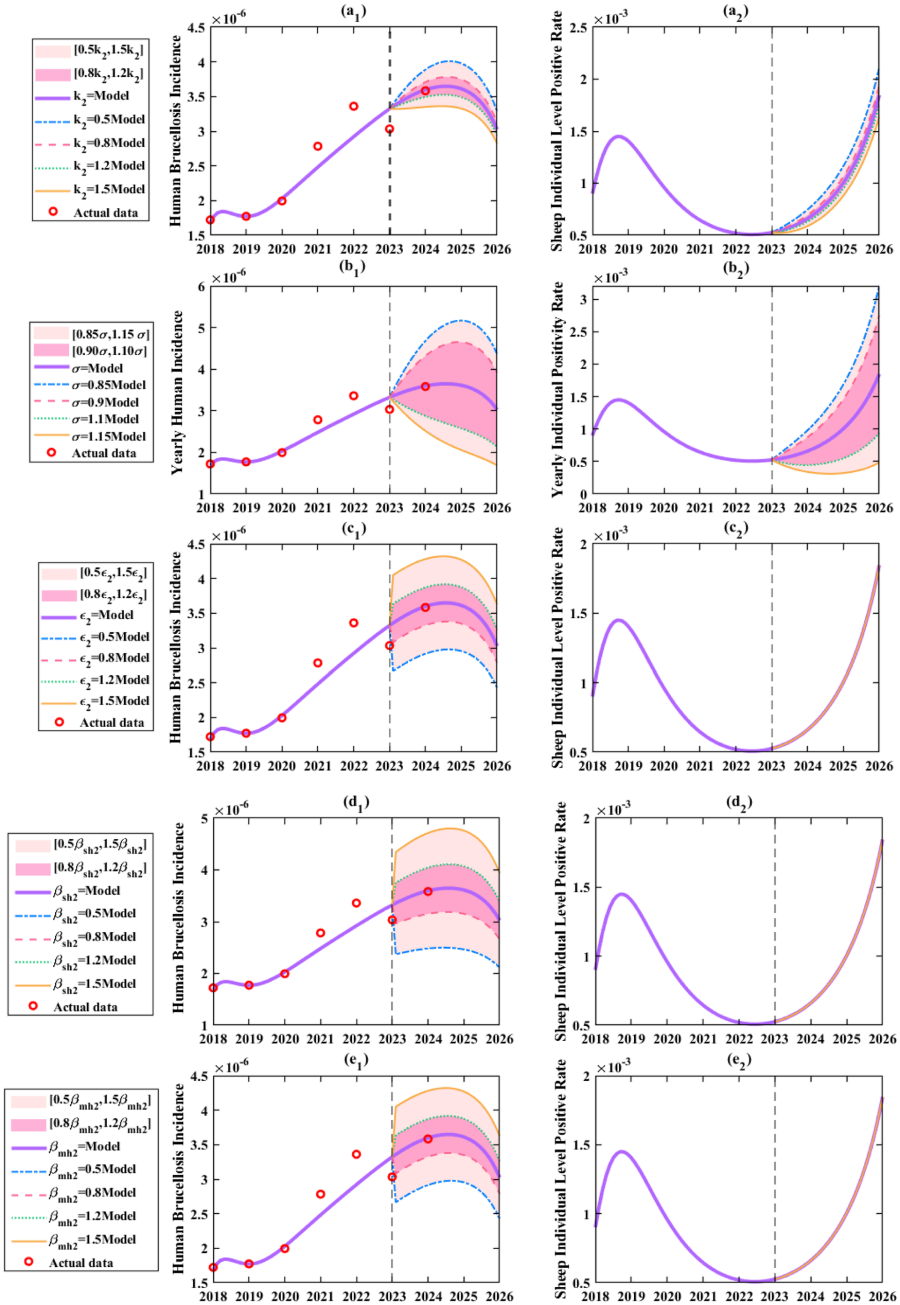
Simulation results evaluating the efficacy of different prevention and control measures: a. Increasing or decreasing breeding link detection efficiency (${k_2}$); b. Increasing or decreasing transportation link detection efficiency ($\sigma$); c. Increasing or decreasing consumption link detection efficiency; d. Increasing or decreasing protective awareness in the breeding link (${\beta _{sh2}}$); e. Increasing or decreasing protective awareness in the consumption link (${\beta _{mh2}}$)

As shown in Fig. 12(a_1_), reducing ${k_2}$ by 50% caused human incidence to first peak at 0.4 per 100,000 before declining，resulting in a 14.3% rise relative to the baseline. Conversely, increasing ${k_2}$ by 50% reduced incidence to 0.29 per 100,000 (decreased by 3.45%) with a downward trend, effectively curbing transmission. Figure 12(b) clearly demonstrated that enhancing ${\rm{\sigma }}$ significantly suppressed all three key indicators. For example, a 15% increase in ${\rm{\sigma }}$ reduced human incidence to below 0.2 per 100,000 (decreased by 66.7%, Fig. 12 (b_1_)) and seroprevalence to below 0.05% (decreased by 72.2%, Fig. 12(b_2_)), whereas a 15% decrease projected incidence to rise to 0.42 per 100,000 (increased by 40%, Fig. 12(b₁)) and seroprevalence to 0.32% (increased by 77.8%, Fig. 12(b_2_)) by 2026.

Figure 12(c) revealed that changes in ${\varepsilon _2}$ strongly influenced human incidence and infected individuals. A 50% increase in ${\varepsilon _2}$ reduced incidence to 0.25 per 100,000 (decreased by 16.7%, Fig. 12(c₁)) by 2026, while a 50% decrease raised it to over 0.35 per 100,000 (a 23.3% increase, Fig. 12(c₁)). Notably, ${\varepsilon _2}$ had no impact on sheep individual level positive rate. Figure 12(d) and 12(e) showed that improving protective awareness (${\beta _{sh2}}$ and ${\beta _{mh2}}$) significantly suppressed all three indicators. Enhancing breeding link awareness (${\beta _{sh2}}$) was particularly effective: a 50% increase projected incidence to drop to 0.2 per 100,000 (decreased by 30%, Fig. 12(d₁)) by 2026, emerging as a highly effective strategy.

#### Optimization of prevention and control strategies

When comprehensively analyzing the impacts of multiple prevention and control measures on brucellosis transmission risk in Zhejiang Province, we focused on evaluating the combined effects of three key factors: detection efficiency across breeding, transportation, and consumption links; the proportion of live sheep vs. mutton transported, and individual protective awareness.

**Fig. 13:**
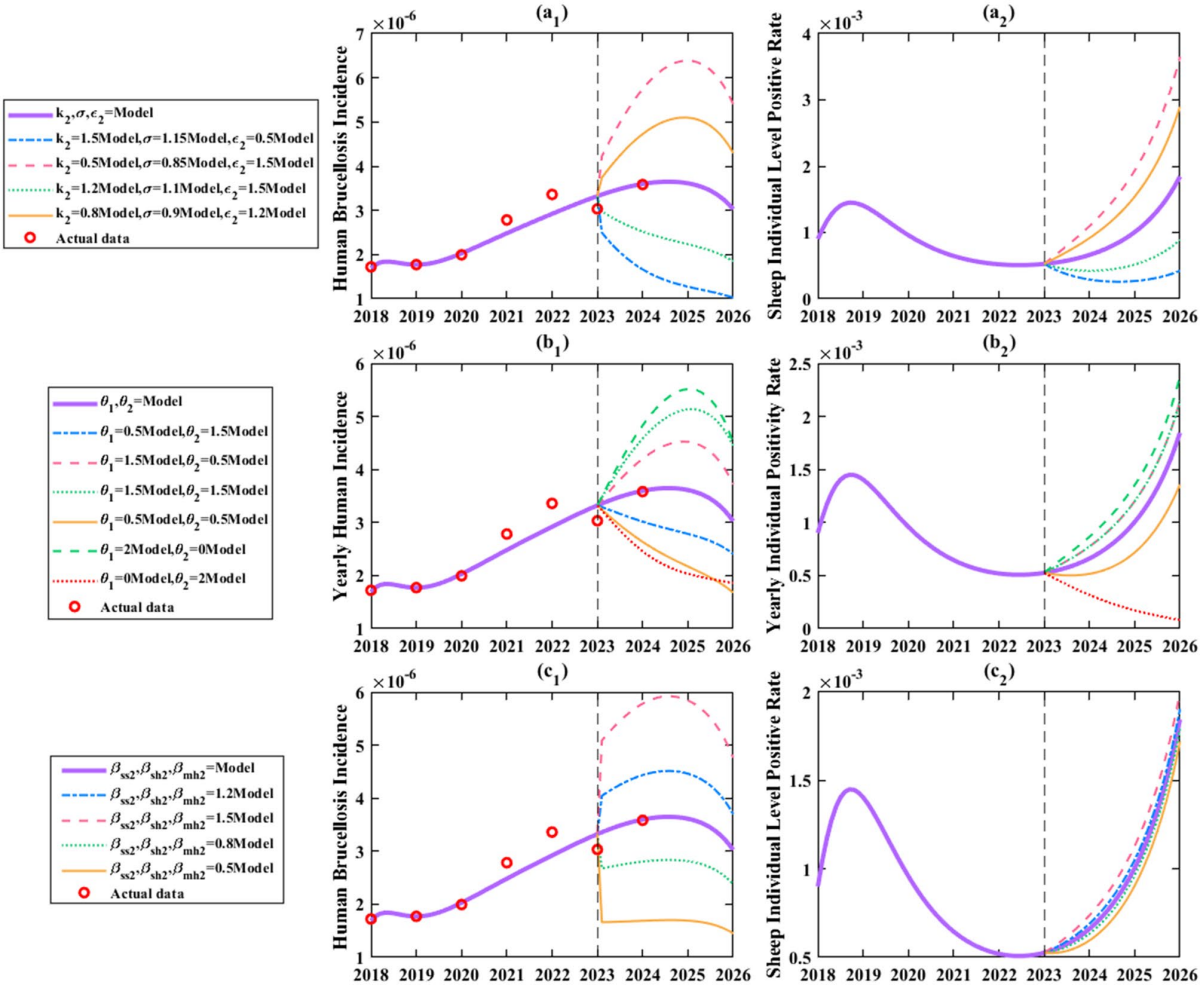
Simulation results of optimized prevention and control strategies: a. Simultaneously increasing or decreasing detection efficiency breeding (${k_2}$), transportation ($\sigma$), and consumption (${\varepsilon _2}$) links; b. Adjusting live sheep and mutton import volumes in combination (both increased/decreased) or individually (increasing live sheep while decreasing mutton, or vice versa) (${\theta _1}$ and ${\theta _2}$); c. Simultaneously increasing or decreasing protection awareness (${\beta _{sh2}}$ and ${\beta _{mh2}}$)

As shown in Fig. 13(a), simultaneously increasing the culling rate (${k_2}$) by 50%, transportation detection rate ($\sigma$) by 15%, and consumption detection rate (${\varepsilon _2}$) by 50% reduced human incidence to a minimum of 0.1 per 100,000 (decreased by 66.7%, Fig. 13(a₁)) with seroprevalence below 0.05% (Fig. 13(a_2_)). Conversely, reducing these rates by 50, 15, and 50% respectively caused incidence to surge to 0.55 per 100,000 (Fig. 13(a₁)) (peak > 0.6 per 100,000), compared with the situation without changing any measures, increased by 83.3% and seroprevalence to 0.35% (increased by 94.4%) with an upward trend. This highlighted that integrated detection efficiency improvements are critical for epidemic control.

Figure 13(b) explored the effects of adjusting live sheep-to-mutton transportation ratios. Reducing both live sheep (${\theta _1}$) and mutton imports (${\theta _2}$) by 50% projected incidence to drop below 0.2 per 100,000 (decreased by 40%, Fig. 13(b₁)) and seroprevalence below 0.15% (decreased by 22.2%, Fig. 13(b_2_)) by 2026, with 91 fewer infections. Increasing both by 50% led to a biphasic incidence trend peaking at 0.43 per 100,000 (increased by 43.3%, Fig. 13(b₁)), 96 more infections, and seroprevalence exceeding 0.2% (increased by 22.2%, Fig. 13(b_2_)). Replacing 50% live sheep imports with mutton imports lowered incidence to < 0.25 per 100,000 (decreased by 20%, Fig. 13(b₁)), whereas substituting mutton with live sheep raised incidence to 0.4 per 100,000 (increased by 33.3%, Fig. 13(b₁)). Banning live sheep imports while doubling mutton imports achieved incidence < 0.2 per 100,000 (decreased by 36.7%, Fig. 13(b₁)) and seroprevalence < 0.02% (decreased by 94.4%, Fig. 13(b_2_)) by 2026. It is evident that the transportation of live sheep exerts a significant impact on the human incidence rate in Zhejiang Province.

Figure 13(c_1_) revealed that enhancing protective awareness and biosecurity measures significantly suppressed human incidence. Increasing awareness in breeding (${\beta _{sh2}}$) and consumption (${\beta _{mh2}}$) links by 50% projected incidence to < 0.15 per 100,000 (decreased by 53.3%) by 2026, while reducing awareness by 50% raised incidence to 0.48 per 100,000 (increased by 60%). Although protective awareness had limited impact on sheep individual level positive rate, its role in lowering human incidence underscored the indispensability of individual protection in epidemic control.

## Discussion

In China, Brucella (*B. melitensis*) is the key pathogen response for large-scale brucellosis outbreaks, with sheep serving as the primary source of infection for human [64, 65]. Although Zhejiang Province is not a major brucellosis-endemic region, the recent sustained increase in human cases highlights significant gaps between current prevention measures and control targets (Fig. 1).

To address this, our study developed a multi-source data-driven transmission dynamic model that integrates population size, breeding scale, live sheep imports, and consumption demand to analyze brucellosis dynamics and effective control strategies in Zhejiang. We identified several critical parameters with significant impacts on human incidence, particularly the effective contact rates between susceptible humans and infected sheep (${\beta _{sh2}}$) or contaminated mutton (${\beta _{mh2}}$), which showed strong positive correlations. Additionally, the positive detection rate of imported live sheep ($\sigma$), the proportion of live sheep imports (${\theta _1}$), and mutton import volume (${\theta _2}$) from Region I also exerted significant effects with its correction gradually increasing from weak to strong over years. Furthermore, the ${\theta _1}$ correlation has surpassed that of both ${\beta _{mh2}}$ and ${\varepsilon _2}$. These findings underscore the critical role of individual protection in epidemic control and confirm that live sheep imports have become a key driver of rising incidence in Zhejiang due to increasing mutton demand [46]. Therefore, to effectively control the spread of the epidemic, efforts should be focused on strengthening the quarantine of cross-provincial live sheep transportation, optimizing the structure of live sheep input, and promoting the tilt of transportation models towards mutton products. At the same time, personal protection in the breeding process and monitoring of the mutton circulation market should be enhanced. A systematic prevention and control network should be constructed through integrated measures targeting input blocking, process protection, and terminal consumption. For example, doubling live sheep imports led to a 73.03% increase in infections, while banning imports reduced infections by 62.75%. Qin et al. (2022) [28] concluded consistent studies on plaque models in Shanxi and Hebei, emphasizing that inter-provincial live livestock transportation is a core driving factor for the spread of brucellosis. This further confirms that even in regions with different epidemic characteristics (such as Class I areas and Class II areas), controlling cross-regional animal movement is a common key link in blocking the transmission chain. Gong et al. (2023) [22] conducted a modeling study on the Ningxia Hui Autonomous Region of China and found that after implementing combined measures including enhanced quarantine, immunization, and compensation for culling, the human incidence rate could be effectively controlled at an extremely low level. Therefore, optimizing transportation strategies by reducing unnecessary live sheep imports and enhancing *σ* is essential. Increasing $\sigma$ by 15% projected incidence to drop below 0.2 per 100,000 with a 90-case reduction by 2026. In addition, this study further analyzed the impacts of ${\theta _1}$ and ${\theta _2}$ on the human incidence rate. The results showed that when the import volume of live sheep was reduced by 50% with a corresponding 50% increase in mutton import volume, the human incidence rate presented a downward trend, and it is projected to drop to 0.25 per 100,000 population by 2026. Conversely, if the proportion of live sheep imported from Region I was increased by 50% while the mutton import volume was reduced by 50%, the human incidence rate would show an upward trend. To quantify the extent of their impacts, this study conducted an extreme scenario simulation: when live sheep imports were completely prohibited and the mutton import volume was doubled, the human incidence rate also exhibited a downward trend. These results fully confirm that the proportion of imported live sheep is a key factor affecting the human incidence rate. In addition, the “National Development Plan for the Livestock and Veterinary Industry during the 14th Five-Year Plan Period” [66] clearly states that “the long-distance cross-regional transportation of live livestock and poultry should be gradually reduced”. It also promotes the model of “centralized slaughtering, cold chain transportation, and cold fresh marketing”. Therefore, optimizing the import structure, i.e., reducing the import volume of live sheep from Region I and increasing the proportion of mutton imports, can serve as an effective strategy for the prevention and control of brucellosis outbreaks in Zhejiang Province.

Regarding prevention strategies, improving the detection efficiency in the transportation link ($$\sigma $$) was more effective than breeding and consumption measures. A 15% increase in $$\sigma $$ reduced human incidence to below 0.2 per 100,000, and the number of infected people by 44.12% in 2026. Combining measures (50% increase in $${k_2}$$, 15% in $$\sigma $$, and 50% in $${\varepsilon _2}$$) achieved a 66.18% infection reduction. Conversely, reducing these rates simultaneously projected a 78.92% infection increase by 2026. Notably, enhancing $${k_2} $$ had limited effects compared to $$\sigma $$, while $${\varepsilon _2} $$ reduced human incidence but not sheep individual level positive rate. Thus, integrated detection efficiency improvements across all links are critical. Governments should implement policies, technical support, and funding to establish long-term control mechanisms and regularly evaluate strategy effectiveness.

Despite providing scientific insights, this study has limitations. Model simplifications (e.g., excluding environmental bacteria and occupational effects, assuming homogeneous human mixing) may underestimate epidemic complexity [67]. Future research should refine models with multi-dimensional factors for improved accuracy. Additionally, real-world implementation of strategies may face challenges like policy enforcement and farmer compliance [68]. Therefore, when formulating prevention and control strategies, it is crucial to fully consider these factors to ensure the effective implementation of the measures. At the same time, due to limitations in data acquisition, the specific impact of certain parameters is difficult to quantify precisely, limiting the accuracy of our predictions regarding future epidemic trends, thereby collecting high-quality data will further refine the model, and enhance its predictive accuracy and real-world applicability.

## Conclusion

This study integrated multi-source data to construct a regional dynamic transmission model for brucellosis in Zhejiang Province, aiming to explore transmission risk characteristics and refining prevention strategies. Through sensitivity analysis and dynamic simulations, we identified the primary drivers of brucellosis transmission and scientifically evaluated current non-immunization-based control measures, proposing targeted optimization recommendations.

Results showed that live sheep imported from Region I serve as the main risk source for brucellosis transmission in Zhejiang. Strengthening quarantine and management of imported sheep is therefore critical to curbing epidemic propagation. Additionally, enhancing detection efficiency across breeding, transportation, and consumption stages is imperative for building effective control barriers. Balancing stable breeding volumes while adjusting live sheep-to-mutton transportation ratios to meet market demand also emerged as a pivotal control tool. The study further emphasized the important role of individual protective awareness in mitigating transmission risks, as heightened public awareness bolsters societal epidemic preparedness.

In conclusion, brucellosis control in Zhejiang requires a multi-dimensional approach, establishing a comprehensive, multi-level prevention and control system. By enhancing quarantine management, improving detection efficiency, optimizing transportation ratios, strengthening individual protection, and implementing integrated measures, the province can effectively mitigate transmission risks and safeguard public health. This research provides scientific evidence and data support for formulating precise control strategies in Zhejiang, while also contributing new perspectives to the study of transmission mechanisms and innovative quantitative evaluation methods in animal disease prevention. With continued data accumulation and model refinement, future research will further optimize strategies and predictive accuracy, offering scientific support for brucellosis control and global public health efforts.

## Appendix A.1: Prediction of the number of human brucellosis cases in Zhejiang Province under different scenarios

**Table:** 

situation	describe	Corresponding parameters	Number of human brucellosis cases		
			2024	2025	2026
	No adjustment	Model	240	242	204
Quantitative assessment of the impact of dominant factors	**S11.** The impact of changes in the volume of live sheep imports	${{\bf{\it{\theta }}}_1}$ = 0	139(−101)	95(−147)	76(−128)
		${{\bf{\it{\theta }}}_1}$ = 0.5Model	188(−52)	166(−76)	138(−66)
		${{\bf{\it{\theta }}}_1}$ = 1.5Model	293(53)	324(82)	276(72)
		${{\bf{\it{\theta }}}_1}$ = 2Model	347(107)	411(169)	353(149)
	**S12.** The impact of changes in the scale of breeding	${{\bf{\it{A}}}_2}$ = 0.5Model	236(−4)	236(−6)	200(−4)
		${{\bf{\it{A}}}_2}$ = 0.8Model	238(−2)	240(−2)	203(−1)
		${{\bf{\it{A}}}_2}$ = 1.2Model	241(1)	245(3)	206(2)
		${{\bf{\it{A}}}_2}$ = 1.5Model	243(3)	248(6)	208(4)
	**S13.** The impact of changes in the quantity of imported lamb meat	${{\bf{\it{\theta }}}_2}$ = 0	214(−26)	201(−41)	155(−49)
		${{\bf{\it{\theta }}}_2}$ = 0.5Model	227(−13)	222(−20)	180(−24)
		${{\bf{\it{\theta }}}_2}$ = 1.5Model	253(13)	263(21)	229(25)
		${{\bf{\it{\theta }}}_2}$ = 2Model	265(25)	283(41)	253(49)
Evaluation of the effectiveness of prevention and control measures	**S21.** The impact of changes in the efficiency of testing during the breeding process	${{\bf{\it{k}}}_2}$ = 0.5Model	259(19)	267(25)	222(18)
		${{\bf{\it{k}}}_2}$ = 0.8Model	247(7)	251(9)	211(7)
		${{\bf{\it{k}}}_2}$ = 1.2Model	233(−7)	234(−8)	198(−6)
		${{\bf{\it{k}}}_2}$ = 1.5Model	223(−17)	223(−19)	190(−14)
	**S22.** The impact of changes in detection efficiency during the transportation process	${\bf{\sigma }}$ = 0.85Model	312(72)	347(105)	294(90)
		${\bf{\sigma }}$ = 0.9Model	288(48)	312(70)	264(60)
		${\bf{\sigma }}$ = 1.1Model	192(−48)	173(−69)	144(−60)
		${\bf{\sigma }}$ = 1.15Model	168(−72)	138(−104)	114(−90)
	**S23.** The impact of changes in consumption process detection efficiency	${{\bf{\it{\varepsilon }}}_2}$ = 0.5Model	194(−46)	198(−44)	164(−40)
		${{\bf{\it{\varepsilon }}}_2}$ = 0.8Model	222(−18)	225(−17)	188(−16)
		${{\bf{\it{\varepsilon }}}_2}$ = 1.2Model	258(18)	260(18)	220(16)
		${{\bf{\it{\varepsilon }}}_2}$ = 1.5Model	285(45)	286(44)	245(41)
	**S24.** The impact of changes in protective awareness during the breeding process	${{\bf{\it{\beta }}}_{{\bf{\it{hs}}}2}}$ = 0.5Model	165(−75)	165(−77)	143(−61)
		${{\bf{\it{\beta }}}_{{\bf{\it{hs}}}2}}$ = 0.8Model	210(−30)	211(−31)	179(−25)
		${{\bf{\it{\beta }}}_{{\bf{\it{hs}}}2}}$ = 1.2Model	270(30)	273(31)	229(25)
		${{\bf{\it{\beta }}}_{{\bf{\it{hs}}}2}}$ = 1.5Model	314(74)	319(77)	266(62)
	**S25.** The impact of changes in consumer protection awareness	${{\bf{\it{\beta }}}_{{\bf{\it{mh}}}2}}$ = 0.5Model	194(−46)	198(−44)	164(−40)
		${{\bf{\it{\beta }}}_{{\bf{\it{mh}}}2}}$ = 0.8Model	222(−18)	225(−17)	188(−16)
		${{\bf{\it{\beta }}}_{{\bf{\it{mh}}}2}}$ = 1.2Model	258(18)	260(18)	220(16)
		${{\bf{\it{\beta }}}_{{\bf{\it{mh}}}2}}$ = 1.5Model	285(45)	286(44)	245(41)
Optimization of prevention and control measures	**S31.** The impact of comprehensive testing: the combined effect of changes in testing efficiency in the breeding, transportation, and consumption stages	${{\bf{\it{k}}}_2}$ = 1.5Model,${\bf{\sigma }}$ = 1.15Model,${{\bf{\it{\varepsilon }}}_2}$ = 0.5Model	111(−129)	85(−157)	69(−135)
		${{\bf{\it{k}}}_2}$ = 0.5Model,${\bf{\sigma }}$ = 0.85Model,${{\bf{\it{\varepsilon }}}_2}$ = 1.5Model	383(143)	428(186)	365(161)
		${{\bf{\it{k}}}_2}$ = 1.2Model,${\bf{\sigma }}$ = 1.1Model,${{\bf{\it{\varepsilon }}}_2}$ = 1.5Model	168(−72)	150(−92)	125(−79)
		${{\bf{\it{k}}}_2}$ = 0.8Model,${\bf{\sigma }}$ = 0.9Model,${{\bf{\it{\varepsilon }}}_2}$ = 1.2Model	315(75)	342(100)	290(86)
	**S32.** The impact of adjusting the transportation mode: the impact of adjusting the proportion of live sheep and mutton transferred	${{\bf{\it{\theta }}}_1}$ = 0.5Model,${{\bf{\it{\theta }}}_2}$ = 1.5Model	201(−39)	187(−55)	162(−42)
		${{\bf{\it{\theta }}}_1}$ = 1.5Model,${{\bf{\it{\theta }}}_2}$ = 0.5Model	280(40)	303(61)	251(47)
		${{\bf{\it{\theta }}}_1}$ = 1.5Model,${{\bf{\it{\theta }}}_2}$ = 1.5Model	306(66)	344(102)	300(96)
		${{\bf{\it{\theta }}}_1}$ = 0.5Model,${{\bf{\it{\theta }}}_2}$ = 0.5Model	176(−64)	146(−96)	113(−91)
		${{\bf{\it{\theta }}}_1}$ = 2Model,${{\bf{\it{\theta }}}_2}$ = 0	322(82)	370(128)	304(100)
		${{\bf{\it{\theta }}}_1}$ = 0,${{\bf{\it{\theta }}}_2}$ = 2odel	164(−76)	136(−106)	125(−79)
	**S33.** The impact of comprehensive personal protective awareness	${{\bf{\it{\beta }}}_{{\bf{\it{ss}}}2}}$,${{\bf{\it{\beta }}}_{{\bf{\it{sh}}}2}}$,${{\bf{\it{\beta }}}_{{\bf{\it{mh}}}2}}$ = 1.2 Model	296(56)	299(57)	250(46)
		${{\bf{\it{\beta }}}_{{\bf{\it{ss}}}2}}$,${{\bf{\it{\beta }}}_{{\bf{\it{sh}}}2}}$,${{\bf{\it{\beta }}}_{{\bf{\it{mh}}}2}}$ = 1.5 Model	386(146)	392(150)	322(118)
		${{\bf{\it{\beta }}}_{{\bf{\it{ss}}}2}}$,${{\bf{\it{\beta }}}_{{\bf{\it{sh}}}2}}$,${{\bf{\it{\beta }}}_{{\bf{\it{mh}}}2}}$ = 0.8 Model	187(−53)	189(−53)	160(−44)
		${{\bf{\it{\beta }}}_{{\bf{\it{SS}}}2}}$,${{\bf{\it{\beta }}}_{{\bf{\it{sh}}}2}}$,${{\bf{\it{\beta }}}_{{\bf{\it{mh}}}2}}$ = 0.5 Model	113(−127)	113(−129)	98(−106)

Note: “+” indicates an increase in the number of cases compared to the predicted value without adjustments; “-” indicates a decrease in the number of cases compared to the predicted value without adjustments

## Appendix A.2: Implementation details of cubic B-spline interpolation parameter estimation

For any time-varying parameter to be smoothed, given its discrete estimated value sequence $\left\{ {\left( {{t_j},{p_j}} \right)} \right\}_{j = 1}^6$ over the baseline years (2018–2023), where (${t_1}$ = 2018, … , ${t_6}$ = 2023), and ${p_j}$ represents the corresponding parameter value. Cubic (fourth-order) B-spline interpolation is employed, and the spline function $S\left( t \right)$ is expressed as:

$$S\left( t \right) = \mathop \sum \limits_{i = 1}^n {c_i}{B_{i,4}}\left( t \right).$$

In the formula, ${c_i}$ represents the control coefficients to be determined, and ${B_{i,4}}\left( t \right)$ is the cubic B-spline basis function defined on the node sequence $\tau = \left\{ {{\tau _1},{\tau _2}, \ldots ,{\tau _{n + 4}}} \right\}$. The basis function ${B_{i,4}}\left( t \right)$ is calculated by the Cox-de Boor recursive formula. First, the zeroth-order (first-order) basis function is defined as:

$${B_{i,1}}\left( t \right) = \left\{ {\matrix{ {1,{\tau _i} \le t \le {\tau _{i + 1}}} \cr {0, else} \cr } } \right..$$

Furthermore, for $k = 2, 3, 4$, the values of higher-order basis functions are recursively calculated according to the following formula:

$$\begin{aligned}{B_{i,k}}\left( t \right) = &{{t - {\tau _i}} \over {{\tau _{i + k - 1}} - {\tau _i}}}{B_{i,k - 1}}\left( t \right) \cr & \quad+ {{{\tau _{i + k}} - t} \over {{\tau _{i + k}} - {\tau _{i + 1}}}}{B_{i + 1,k - 1}}\left( t \right),\end{aligned}$$

when recursion reaches $k = 4$, the resulting function is the required cubic B-spline basis function ${B_{i,4}}\left( t \right)$, which is a piecewise cubic polynomial supported on the interval $\left[ {{\tau _i},{\tau _{i + 4}} } \right]$.

Based on the above node sequence, a $6 \times n$-dimensional design matrix B is constructed, where the element ${B_{ji}} = {B_{i,4}}\left( {{t_j}} \right)$ represents the value of the $j$*th* year on the $ith$ basis function. The control coefficient $c = {\left[ {{c_1}, \cdots ,{c_n}} \right]^T}$ is determined by solving the following least squares problem:

$$min||Bc - p||{^2}, p = {\left[ {{p_1}, \cdots \cdots,{p_6}} \right]^T}.$$

This problem has a unique solution $c = {\left( {{B^T}B} \right)^{ - 1}}{B^T}p$. After solving it, the continuous parameter function $S\left( t \right)$ is obtained.

To facilitate its invocation in the dynamic model and presentation in the main text (Table 4), $S\left( t \right)$ is transformed into a piecewise polynomial representation. Within each sub-interval $\left[ {{t_{j, }} {t_{j + 1}}} \right]$, $S\left( t \right)$ is a cubic polynomial:

$$\begin{aligned}S\left( t \right) = &\:{a_{j0}} + {a_{j1}}\left( {t - {t_j}} \right) + {a_{j2}}{\left( {t - {t_j}} \right)^2} \cr & \quad+ {a_{j3}}{\left( {t - {t_j}} \right)^3}, t \in \left[ {{t_{j, }} {t_{j + 1}}} \right].\end{aligned}$$

The coefficients of each interval $\left\{ {{a_{j0}},{a_{j1}},{a_{j2}},{a_{j3}}} \right\}$ were extracted through the basis function transformation algorithm, and finally the polynomial expression segmented by year as shown in Table 4 was obtained.

This interpolation method ensures that $S\left( {{t_j}} \right) = {p_j}$, and the first and second derivatives of the function are continuous. The fitting curves of all parameters were visually compared with the original discrete estimates (Figs. 4 and 9) to confirm their smoothness and trend consistency, meeting the requirements for subsequent numerical integration of differential equations.

## Data Availability

Data will be made available on request.

## Abbreviations

WHO: World Health Organization
MOA: China’s Ministry of Agriculture and Rural Development
NBS: National Bureau of Statistics
PRCCs: Partial Rank Correlation Coefficients
LHS: Latin hypercube sampling
